# R-GCRED: a retrieval-grounded and calibrated risk estimation for depression prediction and screening recommendation framework

**DOI:** 10.3389/frai.2026.1931579

**Published:** 2026-10-06

**Authors:** Firas Saidi, Marwa Almulla

**Affiliations:** 1 AI and Metaverse Research Center (AIMRC), College of Computer Studies (CCS), University of Technology Bahrain, Salmabad, Bahrain; 2 Department of Psychology, University of Bahrain, Sakhir, Bahrain

**Keywords:** calibration, DAIC-WOZ, decision support, depression analysis, eRisk, explainable AI, LLM, retrieval-augmented generation

## Abstract

Early depression detection from textual narratives has advanced substantially with transformer-based language models and Large Language Models (LLMs). However, reliable screening-oriented decision support requires more than high predictive accuracy. Existing approaches often lack explicit symptom-level grounding, produce poorly calibrated confidence estimates, and provide limited safeguards against uncertainty and unsupported model-generated evidence. This paper introduces R-GCRED, a Retrieval-Grounded and Calibrated Risk Estimation framework for transparent, evidence-grounded, and uncertainty-aware depression risk estimation from text. R-GCRED employs ontology-guided retrieval to constrain LLM-based evidence extraction to clinically relevant symptom facets, producing structured and source-traceable representations that capture symptom identity, polarity, intensity, and supporting textual evidence. A dedicated evidence-verification mechanism further reduces the propagation of unsupported symptom assertions to downstream prediction. The verified evidence is subsequently processed by a dual-head architecture for joint depression-risk classification and severity estimation, followed by probability calibration, uncertainty-aware abstention, and cost-sensitive recommendation modeling. R-GCRED is evaluated on the DAIC-WOZ and CLEF eRisk benchmarks using participant-disjoint evaluation protocols and is compared against general-domain transformers, mental-health-adapted language models, evidence-based methods and LLM-based baselines. Across the evaluated benchmarks, R-GCRED consistently outperforms the compared approaches, indicating that its performance gains extend beyond those obtained against conventional transformer baselines. The framework achieves a Macro-F1 of 0.84, an AUC of 0.89, and an Expected Calibration Error of 0.038, while attaining an AUC of up to 0.90 under partial-observation early-detection settings. Results demonstrate that integrating retrieval-grounded symptom evidence, evidence verification, calibrated prediction, and uncertainty-aware decision modeling yields measurable improvements in predictive discrimination, confidence reliability and explanation traceability over the evaluated existing approaches. These findings establish the computational effectiveness of R-GCRED across the evaluated benchmark settings but do not constitute prospective clinical validation.

## 1 Introduction

Depression is among the most prevalent mental health disorders worldwide and represents a major global public health challenge. According to the World Health Organization, depressive disorders affect more than 280 million individuals globally and are associated with significant social, economic, and health burdens. Early identification of depressive symptoms is important for facilitating timely assessment and appropriate support (World Health Organization, 2025). However, traditional screening procedures often rely on structured assessments or questionnaires administered by trained professionals, which can be resource-intensive and difficult to scale across large populations. In recent years, the increasing availability of digital communication platforms has created new opportunities for analyzing psychological signals expressed through text. Online narratives, such as social media posts, personal diaries, and conversational transcripts, frequently contain linguistic indicators associated with emotional states and mental health conditions. Consequently, Natural Language Processing (NLP) techniques have been widely explored for automated depression-risk detection and mental health analysis (Teferra et al., 2024). Early studies employed traditional Machine Learning (ML) methods using handcrafted linguistic features, sentiment indicators, and psycholinguistic lexicons to identify patterns associated with depressive language (Kumari et al., 2026).

More recently, advances in Deep Learning (DL) and transformer-based language models have significantly improved the ability to capture contextual semantic representations from textual data. Models such as BERT and RoBERTa have demonstrated strong performance across various NLP tasks, including emotion detection and mental health classification (Kumari et al., 2026; More and Mane, 2026). In addition, Large Language Models (LLMs) have shown substantial capabilities in modeling long-range dependencies and complex discourse structures, making them promising computational tools for analyzing psychological narratives (Farruque et al., 2024).

Despite these advances, several important challenges remain when applying language models to mental health analysis tasks. First, many existing approaches rely primarily on latent semantic representations learned from data without explicitly linking predictions to meaningful symptom constructs. As a result, model decisions often lack explicit evidence-level interpretability, limiting their transparency in high-stakes mental-health analysis tasks (Kumari et al., 2026). Second, models trained on specific datasets frequently exhibit domain sensitivity, meaning that performance may degrade when applied across different textual environments, such as transitioning from semi-structured interview transcripts to informal social media narratives. Third, neural classifiers often produce poorly calibrated probability estimates, whereby predicted confidence scores do not accurately reflect observed outcome frequencies. This limitation is particularly relevant to computational risk-estimation frameworks that rely on probability estimates for downstream decision modeling (More and Mane, 2026).

Another important challenge concerns the early detection of depression-related signals. In longitudinal textual settings, evidence associated with depressive symptoms may emerge gradually across multiple interactions rather than within a single document. Systems designed for early-risk estimation must therefore identify meaningful patterns even when only limited textual context is available. Benchmark initiatives such as the CLEF eRisk task have highlighted the importance of developing computational methods capable of detecting mental health risks from partial user timelines (Ibrahimov et al., 2024).

To address these limitations, this paper proposes R-GCRED (Retrieval-Grounded and Calibrated Risk Estimation), a framework for transparent, evidence-grounded, and uncertainty-aware depression-risk estimation from textual narratives. The proposed approach integrates several complementary components designed to improve evidence traceability, predictive robustness, and confidence reliability. First, a structured symptom ontology aligned with established depression-screening constructs provides a knowledge-guided representation of relevant symptom facets. Second, a Retrieval-Grounded Evidence Extraction (R-GEE) mechanism constrains LLM-based evidence extraction by linking textual segments to ontology-defined symptom facets. This process produces structured and source-traceable evidence containing symptom identifiers, evidence polarity and intensity, supporting textual quotations, and concise evidence-grounded rationales. A dedicated evidence-verification mechanism further limits the propagation of unsupported symptom assertions to downstream prediction.

Third, a dual-head prediction architecture jointly estimates depression risk and symptom severity using a multi-task learning formulation. The resulting prediction is subsequently subjected to *post-hoc* probability calibration to improve confidence reliability. An uncertainty-aware abstention mechanism evaluates predictive uncertainty and evidence coverage and allows the framework to withhold a definitive output when confidence is insufficient or available evidence is inadequate. Finally, a cost-sensitive decision component uses calibrated posterior probabilities to investigate asymmetric decision costs within a non-diagnostic, screening-oriented experimental setting. These components are intended as computational mechanisms for benchmark-based depression-risk analysis and should not be interpreted as providing clinical diagnoses.

The effectiveness of R-GCRED is evaluated using two widely used benchmark datasets representing complementary textual settings: semi-structured interview transcripts from DAIC-WOZ and longitudinal social media narratives from CLEF eRisk. Experimental results demonstrate improvements in classification performance, severity-estimation accuracy, and probability calibration compared with general-domain transformer baselines, mental-health-adapted language models, evidence-based approaches, and prompt-based LLM classifiers. Furthermore, the framework is evaluated under partial-observation early-detection conditions, where only progressively increasing fractions of the available longitudinal textual evidence are provided to the model.

Overall, this paper investigates the potential of combining retrieval-grounded symptom evidence, LLM-based evidence extraction, evidence verification, calibrated prediction, and uncertainty-aware decision modeling to develop more interpretable and reliable computational approaches for depression-risk estimation. The evaluation conducted in this study is benchmark-based computational validation and should not be interpreted as evidence of clinical effectiveness or readiness for real-world clinical deployment. Prospective evaluation in representative clinical populations would be required before considering any clinical application. Any potential application of R-GCRED in hospitals, clinical workflows, or patient-facing settings remains a direction for future research and would require prospective clinical validation together with appropriate privacy, safety, ethical, and regulatory evaluation before consideration for real-world use.

The principal contributions of this work are summarized as follows:

- Retrieval-Grounded Evidence Extraction (R-GEE): we propose a knowledge-constrained mechanism that aligns LLM-based evidence extraction with a structured depression symptom ontology, improving evidence traceability and reducing unsupported symptom assertions.
- Dual-Head Multi-Task Learning Architecture: a unified architecture jointly estimates depression-risk classification and symptom-severity scores, enabling complementary predictive modeling from structured symptom-level evidence.
- Calibration-Aware Prediction and Abstention: temperature scaling, uncertainty estimation, and evidence-coverage assessment are integrated to improve probability reliability and enable abstention when predictive uncertainty is high or the available symptom evidence is insufficient.
- Cost-Sensitive Decision Modeling: a cost-sensitive decision mechanism based on calibrated posterior probabilities is investigated for non-diagnostic, screening-oriented recommendation modeling, with asymmetric penalties representing the differing consequences assigned to false-negative and false-positive decisions in the experimental setting.
- Comprehensive Benchmark Evaluation: R-GCRED is evaluated on both DAIC-WOZ and CLEF eRisk, covering interview-based and longitudinal social-media settings and including partial-observation early-detection experiments. These experiments constitute computational benchmark validation rather than prospective clinical validation.

The remainder of this paper is organized as follows. Section 2 reviews the related literature on AI-driven depression detection, explainable AI, and LLM-based mental health screening. Section 3 formulates the problem and defines the objectives of the proposed framework. Section 4 presents the R-GCRED architecture, including retrieval-grounded evidence extraction, dual-head prediction, calibration-aware inference, uncertainty-aware abstention, and cost-sensitive decision modeling. Section 5 describes the experimental setup, datasets, evaluation metrics, validation procedures, and obtained results. Finally, Section 6 concludes the paper, summarizes the main findings, discusses the limitations of the current benchmark-based evaluation, and outlines directions for future research.

## 2 Related work

AI-driven depression prediction has progressed from conventional machine learning and lexicon-based methods toward transformer architectures, Large Language Models (LLMs), multimodal learning, and explainable decision-support systems. Recent systematic reviews confirm that NLP and ML models are increasingly used for depression screening from written and spoken language, but they also highlight persistent concerns regarding generalizability, dataset bias, interpretability, calibration, and clinical validity (Teferra et al., 2024; Fisher et al., 2026). Teferra et al. (2024) reviewed NLP-based depression screening and emphasized methodological limitations, ethical issues, and the need for bias-aware deployment. Similarly, Fisher et al. (2026) conducted a systematic review and meta-analysis of language-based depression detection and showed that model performance varies substantially depending on data source, label quality, language, and modeling strategy. These findings indicate that high predictive accuracy alone is insufficient for clinically reliable decision support.

In clinical-interview settings, recent studies have moved beyond binary classification toward symptom-level estimation and severity prediction. Milintsevich et al. (2023) proposed a text-based symptom prediction approach using DAIC-WOZ interview transcripts, shifting the task from coarse depression classification to the prediction of individual symptom profiles. This symptom-oriented view is important because it improves interpretability and supports more clinically meaningful outputs. Sadeghi et al. (2024) introduced a multimodal framework for depression severity prediction using LLM-derived textual indicators and facial-expression features from E-DAIC, demonstrating the value of combining textual and visual signals for richer assessment. However, these approaches still do not fully integrate retrieval-grounded evidence, calibrated probability estimation, and utility-aware decision mapping within a unified screening framework.

In social-media-based depression detection, recent work has focused on transformer models and LLMs to capture contextual and long-range semantic patterns. Farruque et al. (2024) proposed an LLM-driven semi-supervised approach for depression symptom modeling from social media text, addressing the scarcity of clinically informed symptom-level datasets. Shin et al. (2024) evaluated LLMs for detecting depression from user-generated diary text and validated the potential of semi structured digital narratives as a source for mental health screening. Zhang et al. (2024) developed an NLP-based depression prediction method on Sina Weibo data, demonstrating the relevance of large-scale social media timelines for online risk detection. Shah et al. (2025) further showed that fine-tuned LLMs can improve depression detection from social media platforms, confirming the increasing role of domain-adapted foundation models in mental health analytics. Nevertheless, many of these studies remain primarily prediction-oriented and provide limited mechanisms for clinically grounded reasoning and uncertainty-aware recommendation.

Explainability has also become a central requirement for AI-based depression decision support. Imans et al. (2024) proposed an explainable multi-layer dynamic ensemble framework for depression detection and severity assessment, showing that explainable AI can improve transparency in mental health prediction. Ahmed et al. (2025) developed an explainable AI framework for depression detection and severity classification using SHAP and LIME, demonstrating that *post-hoc* interpretability can provide clinically meaningful insights. Hameed et al. (2025) also investigated explainable AI-driven depression detection from social media data using multiple machine learning models and NLP representations. Despite these advances, most explainability strategies remain *post-hoc* and do not constrain the model's reasoning process before prediction. This limits their ability to guarantee symptom-grounded inference and consistent decision traceability.

Recent domain-specific advances in depression analysis have increasingly moved beyond generic BERT and RoBERTa architectures toward mental-health-adapted representations, symptom-aware modeling, parameter-efficient adaptation, and LLM-based inference. Recent work on DAIC-WOZ has investigated parameter-efficient adaptation for depression severity assessment, demonstrating that prefix-based tuning can effectively adapt pretrained language models to PHQ-8 severity prediction while requiring substantially fewer trainable parameters than conventional full-model fine-tuning (Lau et al., 2023). Complementary symptom-oriented approaches explicitly incorporate depression-screening constructs into the learning process. For example, PHQ-aware architectures integrate symptom-specific descriptive knowledge and textual evidence rather than relying exclusively on latent document-level representations (Zhang et al., 2023). More recently, foundation models have expanded this research direction toward explicit LLM-based depression screening. Teferra et al. (2025) evaluated contemporary LLMs for automated depression screening, illustrating the potential of foundation models for symptom-level mental-health assessment. Similarly, Li et al. (2025) investigated LLM-based depression recognition on DAIC-WOZ by integrating psychological knowledge with multimodal information, further demonstrating the value of domain knowledge in adapting foundation models to depression analysis. Another relevant direction is represented by MentalBERT and MentalRoBERTa, introduced by Ji et al. (2022) as domain-adapted pretrained language models for mental-health NLP. By adapting BERT- and RoBERTa-based representations to mental-health-related textual data, these models provide stronger domain-specific alternatives to generic transformer baselines and are therefore particularly relevant for evaluating depression-detection frameworks. Collectively, these studies indicate that contemporary depression detection increasingly emphasizes domain adaptation, symptom-level reasoning, parameter-efficient learning, and knowledge-enhanced LLM inference. Nevertheless, these advances generally address specific components of the depression-screening pipeline and do not jointly integrate retrieval-grounded symptom evidence, calibrated posterior estimation, explicit uncertainty-based abstention, and cost-sensitive decision mapping within a unified end-to-end decision-support framework.

Despite the increasing sophistication of existing works, the reviewed literature reveals three commonly shared limitations. First, many models remain prediction-centric and do not explicitly anchor outputs to clinically meaningful depression symptom constructs. Second, interpretability is often addressed after prediction through *post-hoc* explanation methods rather than being embedded directly into the inference process through knowledge-constrained reasoning. Third, recent transformer-based and LLM-driven systems rarely integrate calibrated confidence estimation, abstention, and utility-aware recommendation mechanisms into the decision pipeline. The proposed R-GCRED framework addresses these limitations by combining retrieval-grounded symptom evidence extraction, dual-task risk and severity estimation, and calibration-aware decision support within a unified architecture.

Despite the increasing sophistication of existing approaches, the current body of work reveals three fundamental and commonly shared limitations. First, most models remain predominantly prediction-centric, lacking explicit alignment between model outputs and clinically meaningful depression symptom constructs. Second, interpretability is typically addressed in a *post-hoc* manner, relying on external explanation techniques rather than being intrinsically embedded through knowledge-constrained inference mechanisms. Third, recent transformer-based and LLM-driven systems rarely incorporate well-calibrated probability estimation and uncertainty-aware decision-making, limiting their reliability in high-stakes screening scenarios.

The proposed R-GCRED framework is explicitly designed to address these shortcomings by integrating retrieval-grounded symptom evidence extraction, dual-task prediction of risk and severity, and a calibration-aware decision layer within a unified architecture, thereby enhancing interpretability, robustness, and decision reliability.

## 3 Problem definition

Let a subject's textual input be defined as: *X* = {*x*_1_, *x*_2_, …, *x*_*n*_} where each *x*_*i*_ represents an utterance (clinical interview setting) or a post (social media setting). The proposed system produces four outputs:

- Depression likelihood is quantified by Equation 1:

$$\begin{array}{l} \hat{p}=P\left(y=1|X\right) \end{array}$$ (1)

where *y*∈{0, 1} denotes the binary depression label.

- Severity estimate is quantified by Equation 2:

$$\begin{array}{l} ŝ\in \mathbb{R} \end{array}$$ (2)

a continuous severity proxy (e.g., an estimated PHQ-like score when reference severity an notations are available).

- Structured evidence set is represented by the Equation 3:

$$\begin{array}{l} E={\left\{\left(e_{k},r_{k}\right)\right\}}_{k=1}^{K} \end{array}$$ (3)

where *e*_*k*_ denotes a symptom-aligned evidence item extracted from the text and *r*_*k*_ represents the corresponding supporting rationale or quoted snippet.

- Decision tier (Equation 4):

$$\begin{array}{l} d\in \;D \end{array}$$ (4)

representing a screening or triage recommendation selected by minimizing expected decision cost under the calibrated probability estimate. Importantly, the system is designed to support follow-up screening recommendations rather than to provide clinical diagnosis, ensuring alignment with responsible and human-in-the-loop deployment principles.

## 4 Proposed R-GCRED framework

This section presents the proposed R-GCRED framework, a unified architecture for interpretable and reliability-aware depression risk estimation from textual narratives. The framework integrates retrieval-grounded reasoning, structured evidence modeling, and calibration-aware prediction within a principled decision-support pipeline. By combining knowledge-constrained inference with probabilistic calibration and utility-based decision-making, the approach addresses key limitations of existing LLM-based systems. The overall architecture and its components are described in detail in the following subsections.

### 4.1 Architecture overview

The proposed R-GCRED (Retrieval-Grounded, Calibrated Risk Estimation and Decision-Making) framework is a structured, modular architecture (Figure 1) designed to transform unstructured textual narratives into evidence-grounded representations for calibrated and uncertainty-aware depression-risk estimation. The framework supports non-diagnostic, screening-oriented computational analysis and operates through five sequential stages.

**Figure 1:**
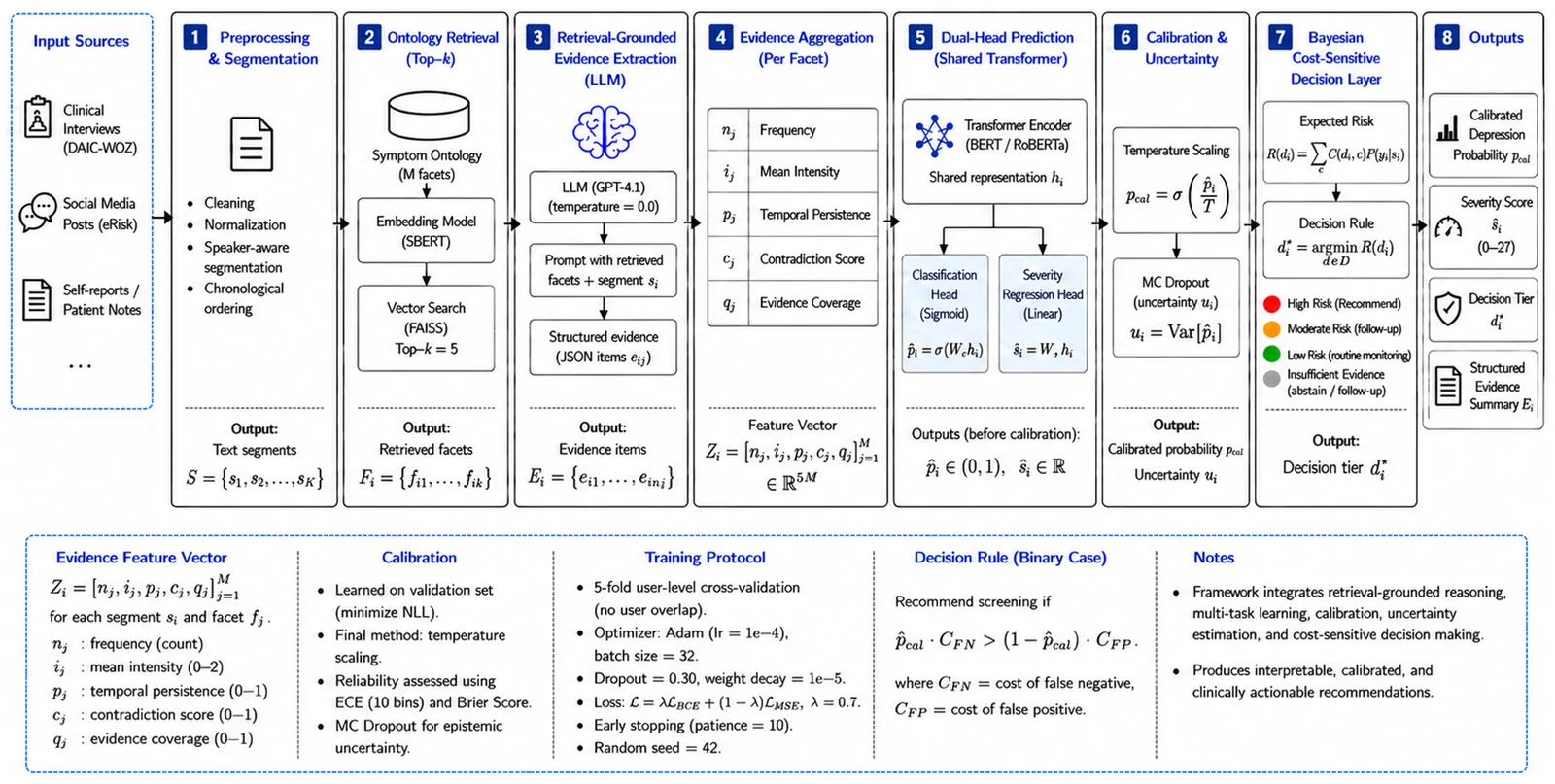
R-GCRED framework pipeline.

First, raw input text either semi-structured clinical interviews (e.g., DAIC-WOZ) or longitudinal social media timelines (e.g., eRisk) is segmented into analysis units using speaker-aware or temporal chunking strategies. Second, a depression symptom ontology aligned with validated screening constructs (e.g., mood disturbance, anhedonia, sleep disruption, fatigue, cognitive impairment) serves as a retrieval anchor. Using embedding-based similarity search, relevant symptom facets are retrieved and injected into a retrieval-grounded prompting mechanism, enabling the large language model to extract structured evidence items consisting of facet identifiers, polarity, intensity, and supporting rationale. Third, extracted evidence is aggregated into a structured feature representation encoding frequency, intensity, temporal persistence, and contradiction patterns. This representation feeds a dual-head predictor that jointly estimates depression likelihood (classification) and severity proxy (regression), encouraging shared symptom-level reasoning. Fourth, *post-hoc* calibration techniques (e.g., temperature scaling) are applied to improve probability reliability, and an abstention mechanism is triggered when uncertainty exceeds predefined thresholds or evidence coverage is insufficient. Finally, a utility-aware decision layer maps calibrated probabilities to screening recommendation tiers by minimizing expected cost, explicitly prioritizing the reduction of false negatives in high-risk contexts. By integrating retrieval grounding, structured evidence extraction, calibration, and decision-theoretic mapping, R-GCRED enhances interpretability, cross-domain robustness, and deployment safety in LLM-based depression screening systems.

#### 4.1.1 Stage 1. Data ingestion and segmentation

The first stage of the R-GCRED framework standardizes heterogeneous textual inputs through domain-aware segmentation strategies. Raw text is first cleaned and normalized, after which it is partitioned into structured analysis units. In clinical interview settings, segmentation is performed at the turn level using speaker-aware parsing to isolate participant utterances from interviewer prompts, thereby reducing artifact bias. In social media contexts, longitudinal timelines are divided into chronological post windows, enabling temporal modeling of symptom persistence and early risk detection. This segmentation step ensures consistent downstream evidence extraction while preserving contextual and temporal structure across domains.

#### 4.1.2 Stage 2. Symptom ontology and retrieval store

A compact ontology Ω defines symptom facets aligned to depression screening constructs (e.g., mood, anhedonia, sleep, appetite, concentration, fatigue, psychomotor changes, guilt/worthlessness, functional impairment). Each facet includes description text, example paraphrases, disambiguation rules (to reduce false positives from slang/irony). The ontology entries are embedded and stored for retrieval.

#### 4.1.3 Stage 3. Retrieval-grounded evidence extraction (R-GEE)

For each segment *x*_*i*_, retrieve top-*m* ontology facets (Equation 5):


$$\begin{array}{l} R_{i}=\text{TopM}\left(sim\left(emb\left(x_{i}\right),emb\left(\Omega \right)\right)\right) \end{array}$$ (5)

Then prompt the LLM with *x*_*i*_+ retrieved facets *R*_*i*_ to produce structured evidence candidates: facet id, polarity (support/neutral/contradict), intensity (0–2) and rationale snippet.

#### 4.1.4 Stage 4. Dual-head prediction (classification + severity)

Aggregate extracted evidence into a feature tensor *Z* (counts, intensities, temporal consistency, contradiction flags). Feed *Z* into (Equations 6, 7):

- Classifier head:

$$\begin{array}{l} f_{c}\left(Z\right)\to p \end{array}$$ (6)

- Regressor head:

$$\begin{array}{l} f_{r}\left(Z\right)\to ŝ \end{array}$$ (7)

This is intentionally not “LLM end-to-end labeling”; instead, the LLM is primarily used for grounded evidence structuring, improving interpretability and domain transfer.

#### 4.1.5 Stage 5. Calibration, abstention

The stage applies *post-hoc* probability calibration to improve the reliability of the predicted depression-risk probabilities. It then evaluates predictive uncertainty and evidence coverage to identify predictions for which the available evidence or model confidence is insufficient. When the predefined uncertainty or evidence-coverage criteria are not satisfied, the framework abstains from issuing a definitive prediction, thereby reducing the risk of overconfident decisions.

#### 4.1.6 Stage 6. Cost-sensitive decision layer

Apply temperature scaling or isotonic regression on validation to calibrate *p* Then:

- If uncertainty exceeds threshold (e.g., entropy or low confidence margin), abstain: output “insufficient evidence requests more data”.
- Otherwise choose decision:

Utility-Based Decision Rule (Equation 8):


$$\begin{array}{l} d^{*}=\arg min_{d\in D}E\left[C\left(d,y\right)|p\right] \end{array}$$ (8)

Expanded Form (Binary Outcome *y*∈{ 0, 1}) (Equation 9):


$$\begin{array}{l} d^{*}=\arg\underset{d\in D}{\min}\left[C\left(d,1\right)p+C\left(d,0\right)\left(1-p\right)\right] \end{array}$$ (9)

Where:

- d^*^ is the optimal decision
- D is the set of possible decisions (e.g., monitor, recommend screening, urgent referral)
- *p* = *P*(*y* = 1∣*X*) is the calibrated probability of depression
- *C*(*d, y*) is the cost function (cost matrix)

In the screening context (Equation 10):


$$\begin{array}{l} C\left(False\;Negative\right)>C\left(False\;Positive\right) \end{array}$$ (10)

meaning that missing a true depression case is penalized more heavily than recommending screening unnecessarily.

Textual inputs are collected from approved sources, including clinical interview transcripts, patient self-reports, diary entries, and longitudinal social media narratives. The system first performs anonymization, text normalization, and context-aware segmentation to generate structured analysis units suitable for downstream processing. Each segment is subsequently transformed into a semantic embedding representation and matched against a clinically informed depression ontology derived from PHQ-aligned and DSM-5 symptom constructs. Using similarity-based retrieval, the most relevant symptom facets are identified and supplied as contextual evidence to a retrieval-grounded evidence extraction module.

The evidence extraction component employs a LLM to generate structured symptom evidence, including symptom category, polarity, intensity, supporting textual quotations, and explanatory rationale. These evidence items are aggregated into a structured feature representation that captures symptom frequency, intensity, temporal persistence, contradiction patterns, and evidence coverage across the observed narrative.

The resulting evidence representation is processed by a dual-head transformer-based prediction model that jointly performs depression classification and severity estimation. The classification branch produces an initial depression risk probability, while the regression branch estimates a PHQ-aligned severity score. To improve the reliability of probability estimates, a *post-hoc* calibration stage based on temperature scaling is applied. Prediction uncertainty is further quantified using Monte Carlo Dropout, enabling an abstention mechanism that suppresses automated recommendations when uncertainty is excessive or evidence coverage is insufficient.

Finally, calibrated probabilities are passed to a cost-sensitive decision layer based on Bayesian decision theory. This layer explicitly accounts for the asymmetric consequences of false-negative and false-positive screening outcomes by minimizing expected decision risk under calibrated posterior probabilities. The resulting outputs, including calibrated depression probability, severity estimate, uncertainty indicators, structured symptom evidence, and recommended follow-up actions, are presented through a healthcare-oriented dashboard designed to support transparent human-in-the-loop screening and clinical decision-making.

### 4.2 Retrieval-grounded evidence extraction (R-GEE)

To ensure clinically aligned reasoning, we introduce a Retrieval-Grounded Evidence Extraction (R-GEE) module that constrains LLM inference using a structured symptom ontology. For each segmented text unit *x*_*t*_, we compute a semantic embedding and retrieve the top-*k* most relevant symptom facets using similarity-based search (Equation 11):


$$\begin{array}{l} F_{t}=\text{Top}K\left(\text{sim}\left(x_{t},f_{j}\right)\right) \end{array}$$ (11)

where *f*_*j*_ denotes a symptom facet defined in the ontology (e.g., depressed mood, anhedonia, sleep disturbance), and sim(·) represents cosine similarity in embedding space.

The retrieved facets *F*_*t*_ are incorporated into a grounded prompting template, guiding the LLM to produce structured evidence rather than unconstrained labels. For each segment, the LLM outputs a structured evidence tuple containing:

- facet_id (ontology-aligned symptom identifier
- polarity ∈{*support, neutral, contradict*}
- intensity ∈{0, 1, 2}
- quote ( ≤ 25-word supporting snippet)
- reasoning (brief explanatory rationale)

This structured output enforces interpretability and traceability. When both supporting and contradicting signals are detected for the same facet, a contradiction flag is raised. Additionally, temporal persistence is tracked across segments to distinguish isolated mentions from recurring symptom patterns. By anchoring inference to retrieved symptom definitions, R-GEE reduces hallucination risk and improves cross-domain robustness.

The R-GEE module employs a fixed, constrained prompt template that is instantiated independently for each text segment. Specifically, for a segment *x*_*t*_, the template receives the segment together with the top-*k* retrieved ontology facets *F*_*t*_, including their symptom identifiers, definitions, clinical descriptions, disambiguation information, and example linguistic expressions available in the retrieval store. The prompt instructs the LLM to operate exclusively as an ontology-grounded evidence extractor rather than as an end-to-end diagnostic classifier. For each retrieved facet, the model determines whether the segment provides supporting, contradictory, or neutral evidence, assigns an intensity value in {0, 1, 2}, extracts a supporting quotation of no more than 25 words when textual evidence is available, and provides a concise evidence-grounded rationale. The output is constrained to a predefined JSON schema containing facet_id, polarity, intensity, quote, and rationale. The model is explicitly instructed not to introduce symptom evidence unsupported by the supplied segment and not to assign facets outside the retrieved candidate set. The prompt structure and extraction rules remain fixed across instances and datasets; only the input segment and its dynamically retrieved ontology context vary. Outputs that fail the predefined schema validation are discarded and regenerated. The complete operational prompt template is provided in Supplementary Appendix S1 to support independent replication of the R-GEE procedure.

The R-GEE module is designed as a modular component and is not intrinsically dependent on GPT-4.1. Within R-GCRED, the LLM acts as a constrained evidence-extraction engine rather than as the final depression classifier. For each input segment, the model receives the retrieved ontology context and maps the available textual evidence to the predefined structured symptom-evidence representation. The downstream evidence aggregation, dual-head classification and severity estimation, probability calibration, uncertainty estimation, abstention mechanism, and cost-sensitive decision mapping operate independently of the selected generative backbone. Consequently, an alternative instruction-following LLM can replace GPT-4.1 without altering the overall R-GCRED architecture, provided that the model can reliably follow the evidence-extraction instructions and generate outputs conforming to the predefined structured schema. This modular separation enables the framework to accommodate different proprietary and open-weight foundation models and reduces architectural dependence on a single LLM provider.

A central requirement of R-GEE is that every generated symptom assertion must remain traceable to the source text. The LLM is therefore constrained to operate as an evidence extractor rather than as an unconstrained clinical reasoner. For each input segment *x*_*t*_, the candidate output space is restricted to the top-*k* ontology facets retrieved for that segment. The prompt explicitly prohibits the introduction of symptoms that are unsupported by *x*_*t*_ and requires the model to assign neutral polarity and intensity 0 whenever the available text does not provide sufficient evidence for either support or contradiction. For every non-neutral evidence item, R-GEE must additionally return a verbatim supporting quotation of no more than 25 words. The retrieved ontology descriptions are used exclusively to define and disambiguate candidate symptom concepts and are not themselves considered evidence that a symptom is present in the source text.

### 4.3 Feature aggregation

The extracted evidence items are aggregated into a structured representation suitable for downstream prediction. For each symptom facet *f*_*j*_, we compute the following statistics:

- Frequency: *n*_*j*_ (number of supporting occurrences)
- Mean intensity: ${\bar{i}}_{j}$ (average severity level)
- Persistence score: *p*_*j*_ (fraction of segments containing the facet)
- Contradiction score: *c*_*j*_ (indicator of mixed or conflicting evidence)

These quantities are combined into a fixed-dimensional feature vector (Equation 12):


$$\phi ={\left[n_{j},{\bar{i}}_{j},p_{j},c_{j}\right]}_{j=1}^{K}$$ (12)

where *K* denotes the number of ontology facets. This structured representation abstracts raw linguistic signals into clinically interpretable symptom features while preserving temporal and contradiction dynamics.

### 4.4 Dual-head prediction

The aggregated feature vector ϕ is fed into a dual-head prediction module that jointly performs classification and severity estimation. The classification head estimates depression likelihood (Equation 13):


$$\begin{array}{l} \hat{p}=\sigma \left(W_{c}\phi +b_{c}\right) \end{array}$$ (13)

where σ(·) is the sigmoid activation function. The regression head predicts a continuous severity proxy (Equation 14):


$$\begin{array}{l} \hat{s}=W_{r}\phi +b_{r} \end{array}$$ (14)

Joint optimization is performed using a weighted multi-task objective (Equation 15):


$$\begin{array}{l} L=\lambda_{1}\;\cdot \;\text{BCE}\left(Y,\hat{p}\right)+\lambda_{2}\;\cdot \;\text{MSE}\left(s,\hat{s}\right) \end{array}$$ (15)

where BCE denotes binary cross-entropy and MSE denotes mean squared error. Multi-task learning encourages shared symptom-level representations to support both binary classification and severity estimation.

### 4.5 Calibration and abstention

Neural classifiers frequently produce overconfident probability estimates, where the predicted confidence does not accurately reflect the true likelihood of the outcome. For example, a model may assign a probability of 0.90 to a prediction that is correct only 70% of the time. Such probability miscalibration can be problematic in healthcare screening applications because downstream recommendations often depend on the estimated risk level rather than the predicted class alone.

To address this limitation, a calibration-aware prediction framework is incorporated into R-GCRED. After model training, a *post-hoc* calibration procedure is applied on a validation dataset using temperature scaling. Let *z* denote the pre-softmax logit produced by the classifier and *T*>0 the learned temperature parameter. The calibrated probability is computed as Equation 16:


$$\begin{array}{l} p_{cal}=\sigma \left(\frac{z}{T}\right) \end{array}$$ (16)

where σ(·) denotes the sigmoid function.

The objective of calibration is not to improve classification accuracy but to ensure that predicted probabilities correspond more closely to observed event frequencies. Consequently, a calibrated probability of 0.80 indicates that approximately 80% of instances assigned that confidence level belong to the positive class. These calibrated probabilities are subsequently used by the decision layer to generate more reliable screening recommendations.

To improve decision reliability, raw probability outputs are *post-hoc* calibrated using temperature scaling (Equation 17):


$$\begin{array}{l} {\hat{p}}_{cal}=\sigma \left(\frac{z}{T}\right) \end{array}$$ (17)

where *z* is the pre-sigmoid logit and *T* is the learned temperature parameter.

In addition to calibration, an uncertainty-aware abstention mechanism is incorporated to prevent unreliable predictions. Prediction uncertainty is estimated using Monte Carlo Dropout, producing a distribution of probability estimates:


$$\begin{array}{l} \left\{p^{\left(1\right)},p^{\left(2\right)},\ldots ,p^{\left(T\right)}\right\} \end{array}$$

The predictive mean and variance are calculated as by Equations 18, 19:


$$\begin{array}{l} \mu =\frac{1}{T}\sum_{t=1}^{T}p^{\left(t\right)} \end{array}$$ (18)

$$\begin{array}{l} \sigma^{2}=\text{Var}\left(p^{\left(t\right)}\right) \end{array}$$ (19)

The system abstains from generating an automated recommendation when the predictive variance exceeds a predefined threshold τ_*u*_ or when evidence coverage falls below a minimum acceptable level. In such cases, the framework returns an “Insufficient Evidence” status and recommends additional information collection or follow-up screening. This mechanism prioritizes reliability and safety by avoiding forced predictions under high uncertainty conditions.

### 4.6 Cost-sensitive decision modeling

The final stage of the proposed R-GCRED framework transforms calibrated prediction probabilities into screening recommendations through a cost-sensitive decision process. Unlike conventional classifiers that rely on a fixed decision threshold (e.g., 0.5), the proposed approach explicitly considers the asymmetric consequences of prediction errors. In mental health screening applications, false-negative predictions may delay further clinical assessment and intervention, whereas false-positive predictions generally result in additional screening procedures. Consequently, the cost associated with missing a potentially depressed individual is typically higher than the cost of recommending additional evaluation for a non-depressed individual.

To address this issue, the framework adopts a Bayesian decision-theoretic formulation in which decisions are selected by minimizing expected decision cost under calibrated posterior probabilities. Let *p*_*cal*_ quantified by the Equation 20:


$$\begin{array}{l} p_{cal}=P\left(y=1|x\right) \end{array}$$ (20)

denote the calibrated probability of depression obtained after the calibration stage, where *y* = 1 indicates the presence of depression and *y* = 0 indicates the absence of depression. Let


$$\begin{array}{l} A=\left\{a_{1},a_{2},\ldots ,a_{m}\right\} \end{array}$$

represent the set of possible screening actions, such as routine monitoring, follow-up screening, or referral for professional assessment.

For each action *a*, the expected decision cost is computed as Equation 21:


$$\begin{array}{l} R\left(a\right)=\sum_{y\in \left\{0,1\right\}}P\left(y|x\right)C\left(a,y\right) \end{array}$$ (21)

where:

*P*(*y*∣*x*) denotes the calibrated posterior probability,

*C*(*a, y*) represents the cost incurred when action *a* is selected while the true outcome is *y*.

The optimal decision is then selected according to the minimum expected risk principle (Equation 22):


$$\begin{array}{l} a^{*}=\arg\underset{a\in A}{\min}R\left(a\right) \end{array}$$ (22)

For the binary screening scenario, the decision rule can be simplified using the costs of false negatives (*C*_*FN*_) and false positives (*C*_*FP*_). The expected risks associated with the two possible decisions are quantified by the Equations 23 and 24 respectively:


$$\begin{array}{l} R\left(\text{Depression}\right)=\left(1-p_{cal}\right)C_{FP} \end{array}$$ (23)

$$\begin{array}{l} R\left(\text{No Depression}\right)=p_{cal}C_{FN} \end{array}$$ (24)

The framework predicts depression (Equation 25):


$$\begin{array}{l} \left(1-p_{cal}\right)C_{FP}<p_{cal}C_{FN} \end{array}$$ (25)

Which can be rewritten by the Equation 26 as:


$$\begin{array}{l} p_{cal}>\frac{C_{FP}}{C_{FP}+C_{FN}} \end{array}$$ (26)

This formulation demonstrates that the decision threshold is not fixed but depends on the relative costs of misclassification. When false negatives are assigned higher cost than false positives (*C*_*FN*_>*C*_*FP*_), the decision threshold decreases, making the system more sensitive to potentially at-risk individuals. This behavior is particularly desirable in depression screening settings where missed cases may have serious clinical consequences. It is important to emphasize that this component is not a formal Multi-Criteria Decision-Making (MCDM) method such as AHP, TOPSIS, ELECTRE, or VIKOR. Instead, it implements a classical cost-sensitive decision rule derived from Bayesian decision theory. The objective is to convert calibrated probabilities into risk-aware screening recommendations while explicitly accounting for the asymmetric consequences of decision errors.

By integrating probability calibration with cost-sensitive decision analysis, the proposed framework produces screening recommendations that are not solely based on classification confidence but also reflect the expected clinical risk associated with different decision outcomes. This design improves the reliability and practical applicability of the framework in real-world mental health screening scenarios.

### 4.7 Evidence verification and hallucination safeguards

To prevent unsupported LLM-generated evidence from propagating to the downstream risk-estimation components, R-GCRED incorporates an evidence-verification stage between R-GEE and evidence aggregation. Let an extracted evidence item be represented as:


$$\begin{array}{l} e_{i}=\left(f_{i},p_{i},a_{i},q_{i},r_{i}\right) \end{array}$$

where *f*_*i*_ denotes the symptom facet, *p*_*i*_ the polarity, *a*_*i*_ the intensity, *q*_*i*_ the supporting quotation, and *r*_*i*_ the rationale. Each generated evidence item is evaluated against the original source segment *x*_*i*_ and its retrieved candidate facet set *F*_*i*_.

An evidence item is accepted only when it satisfies the following conditions: (1) candidate validity, whereby *f*_*i*_∈*F*_*i*_; (2) schema validity, whereby the polarity belongs to {*support, neutral, contradict*} and intensity belongs to {0, 1, 2}; (3) quotation traceability, whereby the reported quotation can be mapped to the original input segment; and (4) evidence consistency, whereby a non-neutral symptom assertion is semantically supported by the quoted source text. The ontology definition itself is never treated as evidence of symptom presence. When the source text is insufficient to establish support or contradiction, the valid representation is neutral with intensity 0.

Evidence items failing verification are assigned a rejection indicator *v*_*i*_ = 0 and are excluded from the evidence vector used by the downstream prediction module. Accepted items receive *v*_*i*_ = 1. Accordingly, the verified evidence set is defined as:


$$E_{\text{verified}}=\left\{e_{i}\in E:v_{i}=1\right\}$$

This verification layer establishes an explicit separation between generation and acceptance: an LLM output is not automatically treated as valid clinical evidence merely because it conforms to the required JSON schema. This design reduces the probability that fabricated or weakly grounded symptom evidence influences depression-risk classification, severity estimation, calibration, or the subsequent screening recommendation.

### 4.8 Algorithmic design

To formalize the operational workflow of the proposed R-GCRED framework, the end-to-end pipeline is expressed as an algorithm that transforms raw textual inputs into calibrated depression risk estimates and screening recommendations. The algorithm describes the sequential interaction between the major components of the framework, including text preprocessing and segmentation, ontology-guided facet retrieval, retrieval-grounded evidence extraction, feature aggregation, dual-head prediction, and calibration-based decision mapping. Given a corpus of textual narratives *X* = {*x*_1_, *x*_2_, ..., *x*_*T*_} the procedure first segments the input into structured analysis units suitable for downstream processing. For each segment, relevant symptom facets are retrieved from the ontology using similarity-based embedding search, and a LLM extracts structured evidence aligned with clinically meaningful constructs. These evidence items are aggregated into a feature representation that captures symptom frequency, intensity, temporal persistence, and contradiction patterns. The resulting feature vector is then processed by a dual-head prediction module to estimate both depression likelihood and severity. Finally, the predicted probability is calibrated and mapped to a screening recommendation using a cost-sensitive decision rule that prioritizes minimizing false negatives. The full computational workflow is summarized in Algorithm 1, which provides a clear and reproducible description of the R-GCRED inference pipeline.

Algorithm 1 R-GCRED*.

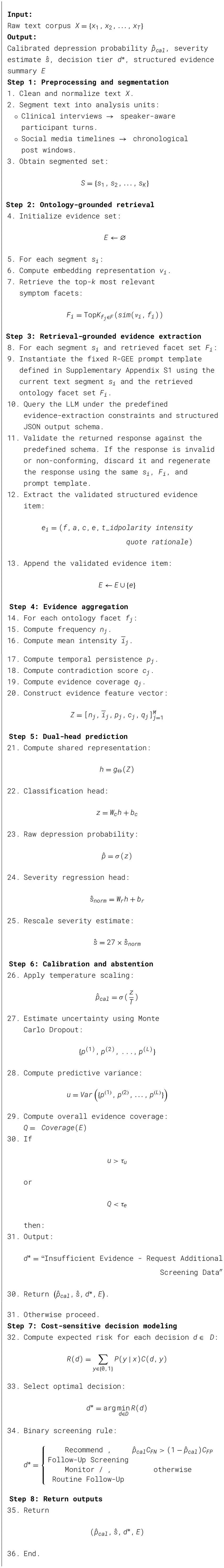

To further strengthen the decision reliability of R-GCRED, an adaptive evidence-weighted refinement algorithm is introduced after the calibration stage. While the calibrated classifier provides a statistically reliable posterior probability, the final screening recommendation may benefit from explicitly considering the strength, persistence, and contradiction level of clinically grounded symptom evidence. Algorithm 2 therefore refines the calibrated probability by combining it with an evidence-weighted risk score derived from ontology-aligned symptom indicators. This additional step improves transparency because the final decision is not based only on the neural prediction output, but also on interpretable symptom-level evidence.

Algorithm 2 **Adaptive evidence-weighted risk refinement.**

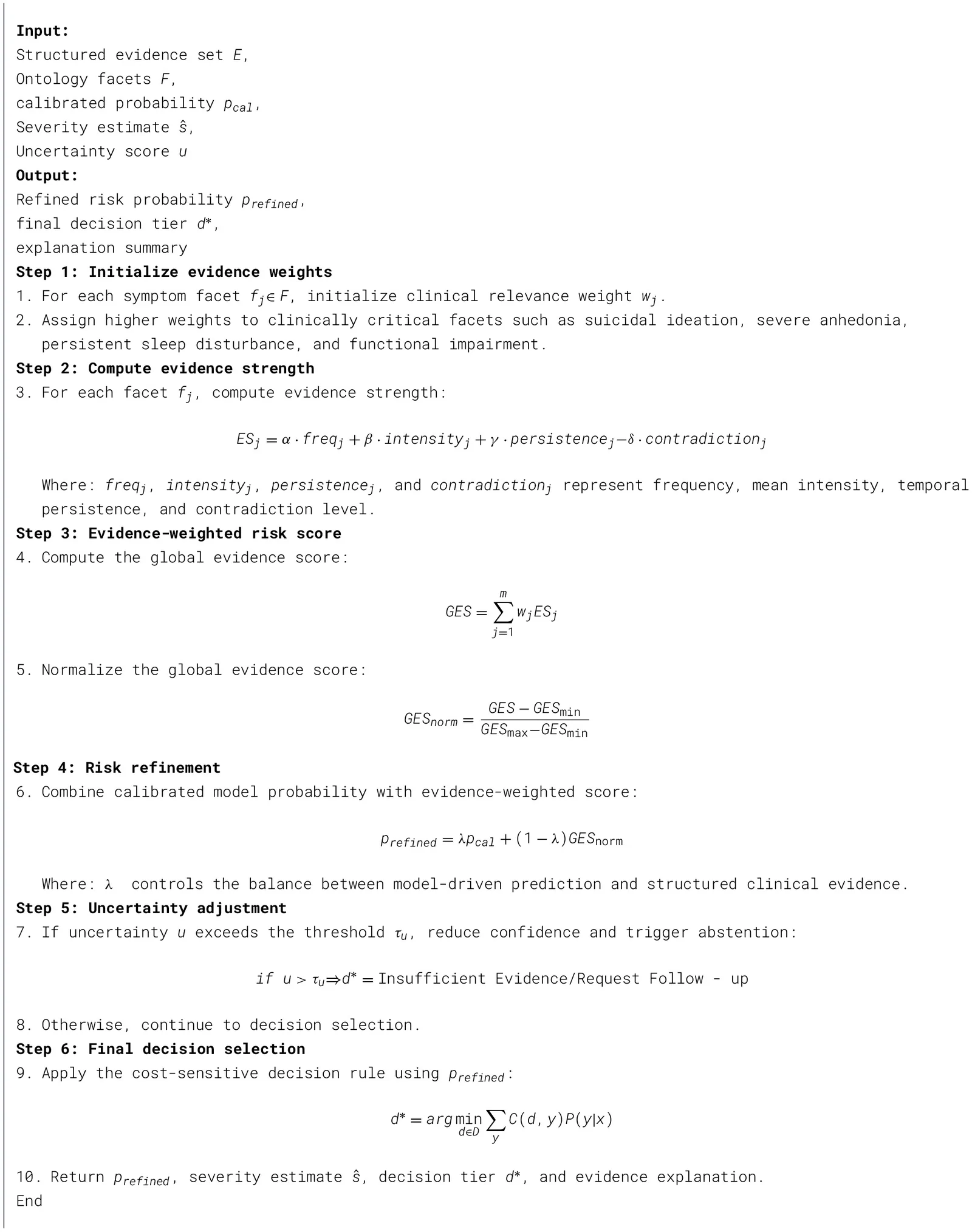

## 5 Experimental evaluation

This section presents the experimental evaluation of the proposed R-GCRED framework. The objective of the evaluation is threefold: (i) to assess the effectiveness of the framework in depression classification, (ii) to evaluate the accuracy of severity estimation, and (iii) to examine the reliability of probability estimates for decision support through calibration analysis. Experiments are conducted across two complementary data settings representing both structured clinical interviews and naturalistic online narratives.

### 5.1 Datasets

To evaluate the robustness and generalization capability of the proposed approach, experiments are performed on two benchmark datasets widely used in depression detection research (see Table 1).

**Table 1:** Sample size and class distribution of the DAIC-WOZ and CLEF eRisk 2025 depression datasets.

Dataset	Evaluation cohort	Depression/positive	Non-depression/control	Positive rate
DAIC-WOZ	189	57	132	30.2%
CLEF eRisk 2025 Task 2 test set	909	102	807	11.2%

This study involved secondary computational analysis of pre-existing benchmark datasets and did not involve prospective participant recruitment, clinical intervention, or direct interaction with human participants by the present authors. Ethical approval and informed-consent procedures associated with the original data collection remain those established by the respective dataset providers. DAIC-WOZ was used in accordance with its controlled research-access conditions, while the eRisk data were processed using the pseudonymized user identifiers provided within the research corpus. The present study did not attempt to identify or re-identify any participant or social-media user. Only textual information required for the reported computational experiments was processed, and no personally identifiable participant information is reproduced in the manuscript. The resulting framework and analyses are intended exclusively for research and non-diagnostic decision-support investigation.

All experimental partitions were defined at the participant/user level. Text segments were generated only after assigning each participant to a partition; individual utterances or posts were never independently distributed across training and evaluation subsets. Consequently, all DAIC-WOZ utterances belonging to the same participant and all longitudinal eRisk posts belonging to the same user remained within a single partition. No participant or user therefore contributed observations to both model training and final testing. This participant-disjoint protocol prevents identity-specific linguistic patterns from leaking across partitions and producing artificially optimistic performance estimates.

- DAIC-WOZ clinical interview dataset

The Distress Analysis Interview Corpus–Wizard of Oz (DAIC-WOZ) is a widely used dataset for automated depression detection from semi-structured clinical interviews. The dataset consists of interactions between human participants and a virtual interviewer, where participants discuss personal experiences, emotional states, and daily life events. Each session is accompanied by depression severity annotations derived from standardized screening instruments. In this work, only the textual transcripts are used. To reduce the influence of interviewer prompts an issue previously reported in the literature we adopt a participant-only protocol in which only participant utterances are considered for model training and evaluation.

DAIC-WOZ contains 189 participant interviews and is distributed into predefined training, development, and test partitions containing 107, 35, and 47 participants, respectively. Using the conventional PHQ-8 cutoff of 10 for binary depression status, the training partition contains 31 depressed and 76 non-depressed participants, the development partition contains 12 depressed and 23 non-depressed participants, and the test partition contains 14 depressed and 33 non-depressed participants. Thus, the corpus exhibits a clinically relevant class imbalance toward the non-depressed class. Only participant utterances were retained in the present text-based experiments; interviewer utterances were excluded to reduce potential prompt-related artifacts.

- CLEF eRisk depression dataset

The CLEF eRisk depression dataset consists of longitudinal Reddit timelines collected for early depression risk prediction. Each user is represented by a sequence of posts arranged chronologically, enabling models to detect depression indicators over time. This dataset reflects real-world language patterns including informal expressions, slang, and narrative variability. Experiments follow the official eRisk evaluation protocol, where performance is evaluated across different fractions of the timeline (10%, 25%, 50%, and 100%) to assess early detection capability.

For longitudinal early-depression evaluation, we use the CLEF eRisk 2025 Task 2: Contextualized Early Detection of Depression collection. The test collection comprises 909 target users, including 102 users in the depression group (11.2%) and 807 control users (88.8%). Each target user is represented by a chronological sequence of writings and associated conversational contexts, enabling evaluation under progressively increasing observation windows. The pronounced class imbalance reflects the naturally skewed prevalence of positive and control cases in the benchmark and motivates the use of discrimination metrics such as AUC in addition to threshold-dependent classification measures. The eRisk data are organized at the user level, and all writings associated with the same target user are maintained within the same experimental partition to prevent cross-user data leakage.

Depression severity in DAIC-WOZ was derived from the PHQ-8 annotations supplied with the dataset and was not generated by the LLM. The PHQ-8 comprises eight symptom items, each scored from 0 to 3, producing a total severity score ranging from 0 to 24. The dataset-provided PHQ-8 total score was used as the continuous reference target for training and evaluating the severity-regression head. For binary depression classification, a PHQ-8 score of 10 was used as the conventional cutoff: participants with PHQ-8 ≥ 10 were assigned to the depressed class, whereas participants with PHQ-8 < 10 were assigned to the non-depressed class. Accordingly, the severity output predicted by R-GCRED should be interpreted as an estimate of the dataset-provided PHQ-8 severity score rather than as an independently established clinical diagnosis.

### 5.2 Deployment

The system exposes secure RESTful and/or FHIR-compatible APIs to allow integration with hospital information systems, electronic health records, telehealth platforms, and mental-health screening applications. The API receives textual input, patient/session metadata, consent status, and optional contextual variables. It returns structured outputs including depression probability, severity estimate, detected symptom facets, supporting evidence snippets, confidence score, abstention flag, and recommended screening tier. Authentication is managed through role-based access control, API keys, OAuth 2.0, or hospital identity-management systems. All API transactions are logged for auditability, traceability, and clinical governance.

Example API outputs may include:

{ “risk_probability”: 0.82, “severity_score”: 17.4, “decision_tier”: “recommend_clinical_screening”, “uncertainty”: 0.11, “abstention”: false, “evidence”: [ { “facet_id”: “DEP_SLEEP_01”, “facet”: “sleep disturbance”, “intensity”: 2, “polarity”: “support”, “supporting_snippet”: “I cannot sleep most nights” } ]}

#### 5.2.1 Memory/retrieval structure

The system maintains a structured retrieval memory composed of three repositories. The first is a symptom ontology store containing clinically aligned depression facets such as mood disturbance, anhedonia, fatigue, sleep disruption, appetite change, guilt, cognitive impairment, psychomotor symptoms, and functional impairment. The second is an embedding-based vector index that stores ontology descriptions, clinical definitions, disambiguation rules, and example linguistic expressions. The third is a temporary session memory that stores segmented user text, retrieved facets, extracted evidence, contradiction flags, and temporal persistence indicators. The retrieval module computes semantic similarity between each text segment and ontology facets, retrieves the top-k relevant symptom constructs, and injects them into the LLM prompting process to constrain reasoning and reduce hallucination.

#### 5.2.2 Runtime orchestration

At runtime, the system follows a controlled orchestration pipeline. First, the input text is validated, anonymized, and segmented into clinically meaningful units. Second, each segment is embedded and matched against the symptom ontology index. Third, the retrieved symptom facets are passed with the text segment to the retrieval-grounded evidence extraction module. Fourth, extracted evidence is normalized into a structured feature vector representing frequency, intensity, persistence, and contradiction. Fifth, the dual-head model generates depression likelihood and severity estimation. Sixth, the calibration module applies temperature scaling or isotonic regression to produce reliable probabilities. Seventh, the uncertainty module determines whether the system should abstain or proceed. Finally, the utility-based decision layer maps the calibrated probability to a risk-aware screening recommendation.

#### 5.2.3 Implementation details

To facilitate reproducibility, the implementation of the proposed R-GCRED framework follows a standardized pipeline. The retrieval-grounded evidence extraction module employs GPT-4.1 through the OpenAI API using deterministic inference (temperature = 0.0, top-p = 1.0, maximum output length = 512 tokens). Semantic retrieval is performed using the Sentence-BERT all-mpnet-base-v2 embedding model (768-dimensional embeddings). Similarity search is conducted using cosine similarity within a FAISS vector index, retrieving the top-k = 5 ontology facets for each text segment.

The prompting strategy follows a constrained template in which the retrieved symptom definitions, clinical descriptions, and example linguistic expressions are injected before the input segment. The LLM is instructed to return a structured JSON object containing: (i) symptom facet identifier, (ii) polarity (support, contradiction, or neutral), (iii) symptom intensity (0–2), (iv) supporting quotation (maximum 25 words), and (v) clinical rationale. Outputs that do not conform to the predefined schema are automatically discarded and regenerated.

The evidence aggregation module computes five statistics for each symptom facet, namely occurrence frequency, average intensity, temporal persistence, contradiction score, and evidence coverage, resulting in a structured feature vector of dimension 5 × M, where M denotes the number of ontology symptom facets.

The dual-head prediction network consists of two fully connected hidden layers (256 and 128 neurons), ReLU activation, batch normalization, dropout rate of 0.30, and Adam optimization with an initial learning rate of 1 × 10–4. Binary cross-entropy and mean squared error are jointly optimized using λ = 0.7 and (1–λ) = 0.3, respectively. Early stopping with patience = 10 epochs is applied to avoid overfitting.

#### 5.2.4 Privacy-preserving API processing

The experiments used the research versions of the DAIC-WOZ and CLEF eRisk datasets made available under their respective access and data-use conditions. Privacy protection was implemented through data minimization, field-level separation, and restricted prompt construction before any GPT-4.1 API request was generated. Specifically, the R-GEE prompt-construction procedure selected only two inputs required for evidence extraction: (i) the current textual segment and (ii) the top-*k* retrieved ontology context associated with that segment. The complete participant record was never transmitted to GPT-4.1. Dataset fields used elsewhere in the experimental pipeline including participant/user identifiers, ground-truth depression labels, PHQ scores where applicable, training/validation/test indicators, cross-validation-fold assignments, file identifiers, and other experimental or administrative metadata were excluded at the prompt-construction stage and remained within the local experimental environment. Participant/user identifiers were retained locally only to associate extracted evidence with the corresponding participant and to support participant-level aggregation and evaluation; they were not inserted into the LLM prompt.

With respect to privacy-sensitive information contained within the textual data themselves, R-GEE operated on the research-release versions of the datasets rather than on original identifiable source records. Where identifying information had been anonymized, pseudonymized, or scrubbed as part of the dataset-provider release process, the framework used the resulting released text and did not reconstruct, recover, or attempt to infer the removed identities. No additional demographic or identifying information was appended to the text before API processing. Each API request was restricted to the minimum segment-level textual content necessary for the corresponding evidence-extraction operation, rather than transmitting a participant's complete dataset record or unnecessary contextual information. The retrieved ontology context contained symptom definitions, clinical descriptions, disambiguation information, and example linguistic expressions and did not contain participant-specific information. Accordingly, information required for dataset management, model development, calibration, and evaluation remained separate from the external LLM inference input, thereby limiting GPT-4.1 exposure to the minimum research text and non-participant-specific ontology information required by R-GEE.

#### 5.2.5 Healthcare workflow integration

The framework is designed to support, not replace, clinical judgment. In a healthcare workflow, R-GCRED can be used as a pre-screening and decision-support tool. A patient may submit textual responses through a mobile application, online portal, or clinical interview system. The system analyzes the input and generates a structured risk report for review by a qualified healthcare professional. Low-risk cases may be flagged for routine monitoring, medium-risk cases may be recommended for standardized screening, and high-risk cases may be escalated for professional assessment according to institutional protocols. The clinician can review the evidence snippets and symptom-level explanations before making any decision. The system also records clinician feedback, which can be used for model monitoring, audit, and future validation.

#### 5.2.6 Edge/cloud inference logic

The system supports hybrid edge-cloud deployment. Lightweight preprocessing, anonymization, segmentation, and initial risk filtering may be executed locally on an edge device, hospital server, or mobile application to preserve privacy and reduce latency. More computationally intensive components, including vector retrieval, LLM-based evidence extraction, dual-head prediction, calibration, and dashboard generation, may be executed in a secure cloud or private institutional server. For sensitive environments, the entire pipeline can be deployed on-premises within a hospital network. The system dynamically selects edge or cloud execution depending on privacy constraints, network availability, computational load, and institutional governance requirements. This architecture enables scalable, privacy-aware, and clinically auditable depression risk estimation.

### 5.3 Evaluation metrics

To comprehensively evaluate model performance, multiple metrics are employed.

- Depression classification performance is evaluated using: Macro-F1 score to account for class imbalance, Area Under the ROC Curve (AUC) measuring ranking performance, sensitivity (Recall), capturing the detection rate of true depression cases. High sensitivity is particularly important in screening applications
- Severity regression metrics: for datasets containing severity labels or proxies, regression performance is evaluated using: Mean Absolute Error (MAE), Root Mean Squared Error (RMSE) and Spearman rank correlation. These metrics measure both prediction accuracy and correlation with reference severity scores.
- Calibration metrics: reliable probability estimates are critical for decision support systems. Calibration quality is evaluated using: Expected Calibration Error (ECE) and Brier Score. These metrics measure the alignment between predicted probabilities and observed outcomes.
- ECE measures the difference between predicted confidence and actual prediction accuracy across confidence bins. Let the prediction probabilities be partitioned into *M* bins ${\left\{B_{m}\right\}}_{m=1}^{M}$. The ECE is defined by the Equation 27 as:

$$\begin{array}{c} \text{ECE}=\sum_{m=1}^{M}\frac{|B_{m}|}{n}|\text{acc}\left(B_{m}\right)-\text{conf}\left(B_{m}\right)| \end{array}$$ (27)

where:

- *n* is the total number of samples
- ∣*B*_*m*_∣ is the number of predictions in bin *m*
- *acc*(*B*_*m*_) is the average accuracy of predictions in bin *m*
- *conf*(*B*_*m*_) is the average predicted confidence in bin *m*

Lower ECE values indicate better probability calibration and more reliable confidence estimation.

The Brier Score evaluates the accuracy of probabilistic predictions by measuring the mean squared difference between predicted probabilities and true labels (Equation 28):


$$\text{Brier Score=}\frac{\text{1}}{\text{n}}\sum_{\text{i=1}}^{\text{n}}({\hat{\text{p}}}_{\text{i}}-{\text{y}}_{\text{i}}{)}^{\text{2}}$$ (28)

where:

- *n* is the number of samples
- ${\hat{p}}_{i}\;$is the predicted probability for sample *i*
- *y*_*i*_∈{0, 1} is the ground-truth label

A lower Brier Score indicates better calibrated and more accurate probabilistic predictions.

- Early detection metrics: for the eRisk dataset, early detection performance is measured using: AUC across timeline fractions and early-risk utility metrics defined in the CLEF eRisk evaluation protocol.

### 5.4 Model validation and robustness assessment

To reduce the risk of overfitting, quantify model uncertainty, and assess the robustness of the proposed R-GCRED framework, multiple complementary validation procedures were employed. These procedures evaluate predictive stability, statistical significance, generalization capability, and component-level contribution.

The experimental workflow strictly separates model fitting, model selection, probability calibration, and final evaluation. The training partition is used to optimize the parameters of the R-GCRED prediction network. Validation data are used for hyperparameter selection and early stopping. Probability calibration is performed only after the predictive model parameters have been fixed, with the temperature parameter estimated from held-out calibration observations by minimizing calibration loss. Neither model parameters nor calibration parameters are optimized using final test observations. Once model selection and calibration are completed, the resulting configuration is frozen and applied to the test partition for final computation of classification, severity, calibration, uncertainty, and decision-support metrics.

#### 5.4.1 Stratified K-fold cross-validation

All experiments were conducted using stratified five-fold cross-validation to ensure reliable estimation of model performance while preserving class distributions across folds. Let the dataset *D* be partitioned into *K* = 5 mutually exclusive folds (Equation 29):


$$\begin{array}{c} D={⋃}_{k=1}^{K}D_{k} \end{array}$$ (29)

where: *D*_*i*_∩*D*_*j*_ = ∅∀*i*≠*j*

At each iteration, one fold was used for testing while the remaining *K*−1 folds were used for training. The average performance metric *M* was computed based on Equation 30:


$$\begin{array}{c} \bar{M}=\frac{1}{K}\sum_{k=1}^{K}M_{k} \end{array}$$ (30)

and the corresponding standard deviation was calculated based on Equation 31:


$$\sigma_{M}=\sqrt{\frac{1}{K-1}\sum_{k=1}^{K}(M_{k}-\bar{M}{)}^{2}}$$ (31)

where *M*_*k*_denotes the metric value obtained on fold *k*.

This procedure provides an estimate of the model's expected generalization performance while reducing dependence on a single train-test split.

No observations from the held-out evaluation fold were used for model fitting, hyperparameter selection, early stopping, probability calibration, or threshold selection in the corresponding iteration. Probability calibration was performed using validation data within the model-development data, and all uncertainty-, evidence-coverage-, and cost-related decision parameters were determined without access to the held-out evaluation fold. Only after completion of these model-development procedures was the corresponding held-out fold used to calculate classification, severity-estimation, and calibration metrics. Consequently, the reported results represent aggregated performance across five mutually exclusive held-out evaluations and not performance obtained from a single fixed train–test split.

To improve transparency regarding class composition and experimental partitioning, Table 2 reports the number of depression-positive and depression-negative/control cases associated with the training, validation, calibration, and test/evaluation data. All counts are reported at the participant/user level rather than at the level of individual utterances, segments, or posts.

**Table 2:** Positive and negative cases in the training, validation, calibration, and test/evaluation data.

Dataset	Partition/experimental role	Depression-positive (n)	Depression-negative/control (n)	Total (N)	Use in R-GCRED
DAIC**-**WOZ	Official training partition	**31**	**76**	**107**	Source benchmark training data
	Official development/validation partition	**12**	**23**	**35**	Validation and model-development data
	Official test partition	**14**	**33**	**47**	Source benchmark test data
	**Complete cohort**	**57**	**132**	**189**	Participant-level five-fold CV cohort
	Model-development portion per CV iteration	**45–46**	**105–106**	**151–152**	Model development within five-fold CV
	**Held-out evaluation fold per CV iteration**	**11–12**	**26–27**	**37–38**	**Held-out cross-validation evaluation only**
CLEF eRisk 2025 Task 2	**Held-out evaluation cohort**	**102**	**807**	**909**	Longitudinal early-detection evaluation
	10% timeline observation	**102**	**807**	**909**	Evaluation using first 10% of each user's timeline
	25% timeline observation	**102**	**807**	**909**	Evaluation using first 25% of each user's timeline
	50% timeline observation	**102**	**807**	**909**	Evaluation using first 50% of each user's timeline
	100% timeline observation	**102**	**807**	**909**	Full-timeline evaluation

For DAIC-WOZ, the complete cohort comprised 189 participants, including 57 depression-positive and 132 depression-negative participants. The official benchmark partitions comprised 107 training participants (31 positive and 76 negative), 35 development/validation participants (12 positive and 23 negative), and 47 test participants (14 positive and 33 negative). Depression-positive status was defined using the standard PHQ-8 threshold of ≥ 10.

The final DAIC-WOZ results reported in this study were obtained using stratified five-fold cross-validation at the participant level, rather than from a single fixed train-test split. In each iteration, one fold was held out for evaluation and the remaining four folds constituted the model-development portion, consistent with the cross-validation procedure described in this study. Consequently, approximately 151–152 participants (45–46 positive and 105–106 negative) were available for model development in each iteration, whereas 37–38 participants (11–12 positive and 26–27 negative) constituted the corresponding held-out evaluation fold.

*Post-hoc* probability calibration was performed on validation data, as specified in the calibration procedure. Thus, calibration data did not constitute an additional independent participant cohort; the validation observations used for calibration retained their validation-set identity and were not counted as an additional set of participants.

Counts are reported at the participant/user level rather than at the level of individual utterances, segments, or posts. For DAIC-WOZ, depression-positive status was defined as PHQ-8 ≥10, whereas PHQ-8 < 10 represented the depression-negative class. The complete DAIC-WOZ cohort comprised 189 participants (57 positive and 132 negative). Under participant-level stratified five-fold cross-validation, approximately 151–152 participants (45–46 positive and 105–106 negative) constituted the model-development portion and 37–38 participants (11–12 positive and 26–27 negative) constituted the held-out evaluation fold in each iteration. The small variation results from integer allocation of the class counts across five folds. The manuscript specifies that *post-hoc* probability calibration was performed using validation data; therefore, the calibration row does not represent an additional independent cohort and its participants must not be added again when calculating the total sample size.

For CLEF eRisk 2025 Task 2, the held-out longitudinal evaluation cohort comprised 909 users (102 depression-positive and 807 controls). The 10%, 25%, 50%, and 100% conditions correspond to progressively increasing fractions of the chronological textual history available for the same evaluation users, rather than separate participant datasets; consequently, the class composition remains 102 positive and 807 control users at each observation point. This is consistent with the longitudinal early-detection protocol described in the manuscript.

To provide a more detailed account of the data allocation within each cross-validation iteration, Table 3 reports the separate numbers of depression-positive and depression-negative participants used for training, validation, and *post-hoc* probability calibration across the five folds. The validation subset was also used for calibration; therefore, the calibration counts refer to the same participants and do not represent an additional independent data partition.

**Table 3:** Fold-specific distribution of depression-positive and depression-negative cases used for training, validation, and calibration in DAIC-WOZ.

Cross-validation iteration	Training positive	Training negative	Validation positive	Validation negative	Calibration positive^*^	Calibration negative^*^
Fold 1	39	92	6	14	6	14
Fold 2	39	92	6	14	6	14
Fold 3	40	91	6	14	6	14
Fold 4	40	91	6	14	6	14
Fold 5	40	92	6	14	6	14

^*^Positive and negative denote depression-positive and depression-negative participants, respectively. The validation/calibration observations are a subset of the model-development portion reported in Table 2. Calibration does not represent an additional or overlapping independent partition: *post-hoc* probability calibration was performed using the corresponding validation subset.

Within each cross-validation iteration, the participant-disjoint model-development portion reported in Table 2 was further allocated into training and validation/calibration roles using stratified sampling to preserve the depression-positive/depression-negative distribution as closely as possible. The training subset contained 39–40 depression-positive and 91–92 depression-negative participants, depending on the outer fold, while the validation subset contained six depression-positive and 14 depression-negative participants in each iteration. The validation subset was used for model-development decisions and subsequently for *post-hoc* probability calibration; therefore, calibration did not introduce an additional participant subset, and the same six positive and 14 negative validation cases constituted the calibration data in each iteration. The corresponding held-out evaluation fold remained separate from these model-development data and was not used for model fitting or probability calibration. Table 3 reports the complete fold-specific class distribution for these roles.

#### 5.4.2 Bootstrap validation

To assess performance stability and estimate confidence intervals, bootstrap resampling was performed using *B* = 1, 000 iterations. For each bootstrap iteration, a new dataset was generated by sampling with replacement from the original dataset.

Let: θ^(*b*)^ denote the performance metric obtained in bootstrap iteration *b*.

The bootstrap estimate is computed based on the Equation 32:


$$\begin{array}{c} {\hat{\theta }}_{boot}=\frac{1}{B}\sum_{b=1}^{B}\theta^{\left(b\right)} \end{array}$$ (32)

The standard bootstrap error is described in the Equation 33:


$$\begin{array}{c} SE_{boot}=\sqrt{\frac{1}{B-1}\sum_{b=1}^{B}{\left(\theta^{\left(b\right)}-{\hat{\theta }}_{boot}\right)}^{2}} \end{array}$$ (33)

The 95% confidence interval was estimated using percentile-based bounds:


$$\begin{array}{c} CI_{95\%}=\left[\theta_{2.5\%},\theta_{97.5\%}\right] \end{array}$$ (34)

Bootstrap analysis was performed for Macro-F1, AUC, Expected Calibration Error (ECE), and Brier Score.

This validation strategy provides an estimate of model stability and quantifies uncertainty associated with performance measurements.

#### 5.4.3 Permutation (randomization) testing

To determine whether the observed predictive performance could occur by chance, a permutation test was conducted following the principles of label randomization validation.

The null hypothesis is defined the Equation 35 below:


$$\begin{array}{c} H_{0}:X⊥Y \end{array}$$ (35)

where the feature representation *X* is independent of the class labels *Y*.

Class labels were randomly shuffled *N* = 1, 000 times while preserving the original feature distribution. For each permutation, the complete training and evaluation pipeline was repeated, producing a performance statistic *T*_*i*_.

The empirical *p*-value was computed as:


$$\begin{array}{c} p=\frac{1+\sum_{i=1}^{N}I\left(T_{i}\geq T_{obs}\right)}{N+1} \end{array}$$ (36)

where:

- *T*_*obs*_ denotes the observed model performance (e.g., AUC),
- *I*(·) is the indicator function.

A statistically significant result (*p* < 0.05) indicates that the observed performance is unlikely to arise from random label assignments and therefore reflects meaningful relationships within the data.

#### 5.4.4 Ablation-based validation

To quantify the contribution of individual architectural components, systematic ablation experiments were conducted.

Let: *P*_*full*_ denote the performance of the complete R-GCRED framework and *P*_−_*c* the performance obtained after removing component *c*.

The contribution of component *c* is measured by the Equation 37 as:


$$\begin{array}{c} \Delta_{c}=P_{full}-P_{-c} \end{array}$$ (37)

where larger values of Δ_*c*_ indicate greater influence on overall system performance. Three principal ablation configurations were evaluated:

1. Removal of ontology-guided retrieval.
2. Removal of calibration and uncertainty estimation.
3. Removal of the dual-head classification–severity prediction module.

This analysis enables identification of the architectural elements responsible for improvements in predictive performance and calibration reliability.

The combination of stratified cross-validation, bootstrap resampling, permutation testing, and ablation analysis provides complementary evidence regarding model robustness, statistical significance, stability, and generalization capability. Cross-validation estimates predictive performance under different data partitions, bootstrap analysis quantifies uncertainty, permutation testing verifies that performance exceeds chance level, and ablation studies validate the contribution of each architectural component. Collectively, these validation procedures strengthen confidence in the reliability and reproducibility of the proposed R-GCRED framework.

### 5.5 Results and analysis

This section presents the empirical evaluation of the proposed R-GCRED framework across multiple datasets and experimental settings. The objective of the analysis is to assess the effectiveness of the framework in depression classification, severity prediction, probability calibration, and early risk detection. Quantitative results are reported using the evaluation metrics described in Section 6.3 and are compared against several strong baselines, including transformer-based classifiers and prompt-based large language models.

In response to the rapid evolution of language modeling for computational mental-health analysis, the experimental comparison was extended beyond conventional BERT and RoBERTa models to include domain-adapted and recent depression-specific approaches. MentalBERT and MentalRoBERTa were selected as representative mental-health-domain language models because their pretraining incorporates textual data from mental-health communities and therefore provides a stronger domain-specific comparison than generic transformer encoders. For depression severity estimation on DAIC-WOZ, a parameter-efficient prefix-tuning approach was additionally considered because this class of methods has demonstrated strong performance for PHQ-oriented severity prediction on the same benchmark. Recent PHQ-aware symptom models and LLM-based depression-detection approaches were also examined in the literature comparison. However, published results obtained using different corpora, task definitions, label spaces, sampling strategies, or train/test partitions were not directly merged with the present numerical benchmark, since such comparisons would confound architectural differences with dataset and protocol differences. Direct experimental comparisons reported below therefore use a common evaluation protocol wherever model reproduction is technically and methodologically compatible, while non-equivalent recent approaches are discussed separately as contextual SOTA references.

The analysis is organized into four main parts. First, we evaluate the classification performance of R-GCRED on the DAIC-WOZ clinical interview dataset to assess its ability to detect depression from conversational language. Second, we examine the framework's capability to estimate depression severity using regression-based evaluation metrics. Third, we analyze the reliability of predicted probabilities through calibration metrics, which are critical for downstream decision-making processes. Finally, we investigate the early detection performance of the framework on the CLEF eRisk dataset to evaluate its ability to identify depression signals under partial information conditions. In addition to reporting quantitative metrics, we provide an ablation analysis to quantify the contribution of the major architectural components of the R-GCRED framework, including ontology-guided retrieval, the dual-head prediction module, and the calibration layer. This analysis helps clarify how each component contributes to the overall performance and reliability of the system.

#### 5.5.1 Depression classification performance

The classification performance of the proposed R-GCRED framework is evaluated on the DAIC-WOZ dataset and compared against several baseline approaches. The results are summarized in Table 4, which reports Macro-F1, AUC, and sensitivity scores for each model.

**Table 4:** Depression classification performance on the DAIC-WOZ dataset.

Model	Model category	Macro-F1 ↑	AUC ↑	Sensitivity ↑
BERT Fine-tuned	General-domain transformer	0.74	0.80	0.71
RoBERTa Fine-tuned	General-domain transformer	0.76	0.82	0.73
MentalRoBERTa (Ji et al., 2022)	Mental-health-adapted transformer	0.80	0.86	0.78
Prompt-only LLM	Foundation-model baseline	0.78	0.83	0.75
Evidence + Logistic Regression	Evidence-based baseline	0.79	0.84	0.76
**R-GCRED (Proposed)**	**Retrieval-grounded calibrated framework**	**0.84**	**0.89**	**0.82**

↑ indicates that higher values represent better performance.

As shown in Table 3 and Figure 2, the proposed R-GCRED framework achieves the highest performance across all classification metrics, outperforming both general-domain transformer baselines and the mental-health-adapted MentalRoBERTa models. In particular, Macro-F1 increases from 0.74–0.80 across the transformer baselines to 0.84 with R-GCRED, while AUC improves from 0.80–0.86 to 0.89. Sensitivity similarly increases from 0.71–0.78 to 0.82, indicating an improved ability to correctly identify true depression cases. Notably, the improvement over MentalRoBERTa, the strongest domain-adapted transformer baseline, demonstrates that mental-health-specific pretraining alone does not account for the performance gains achieved by R-GCRED. These results suggest that retrieval-grounded evidence extraction provides additional value by guiding the language model toward clinically meaningful symptom evidence. By anchoring predictions in ontology-defined symptom constructs, R-GCRED reduces reliance on superficial lexical patterns and instead emphasizes linguistically expressed indicators associated with depressive states.

**Figure 2:**
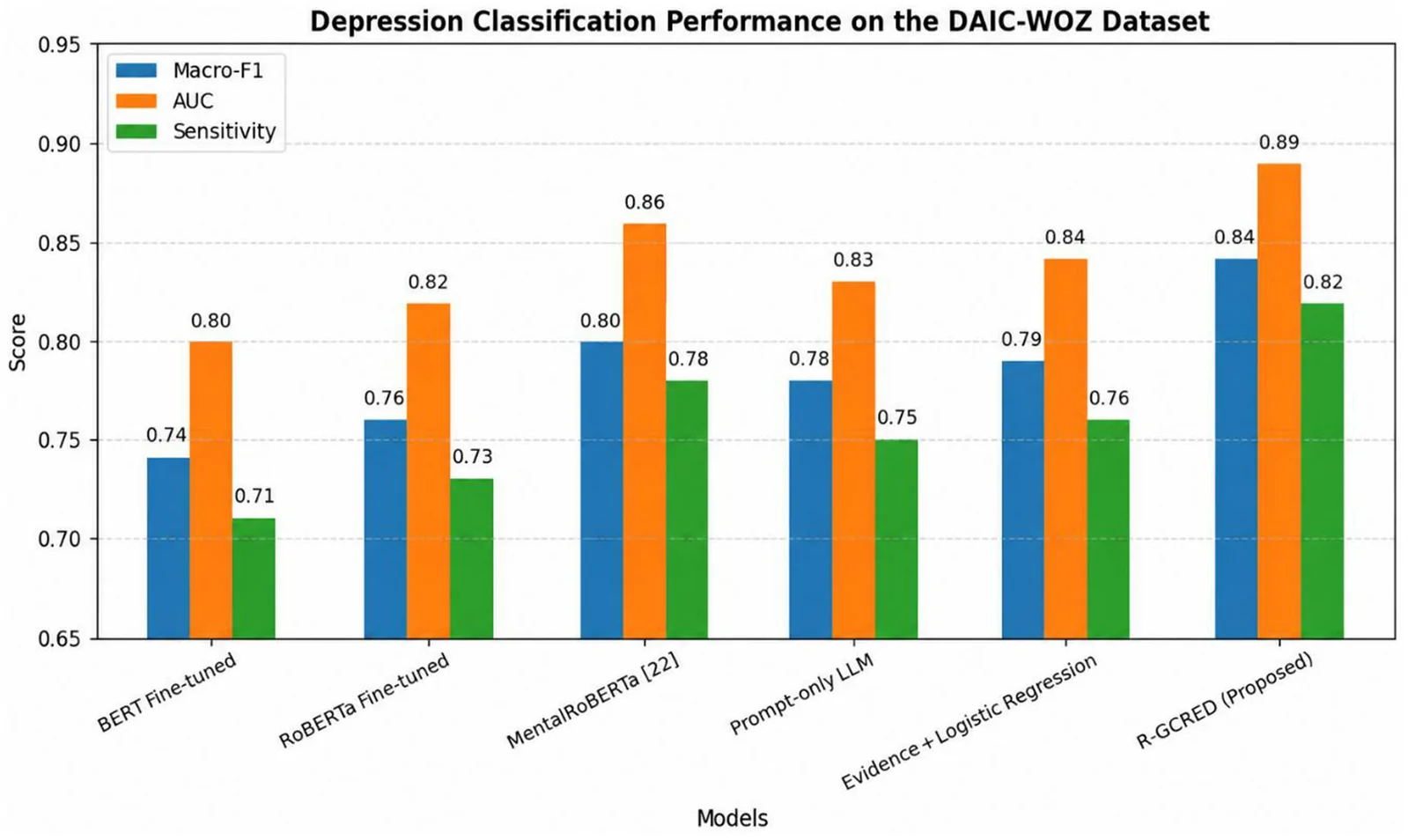
Depression classification performance comparison on the DAIC-WOZ.

#### 5.5.2 Severity estimation performance

In addition to binary depression classification, the proposed framework supports severity estimation through its dual-head prediction architecture. The results are reported in Table 5 and Figure 3, where performance is evaluated using MAE, RMSE, and Spearman rank correlation.

**Table 5:** Severity prediction performance.

Model	Model category	MAE ↓	RMSE ↓	Spearman ρ ↑
**BERT regression**	General-domain transformer	3.52	4.67	0.61
**RoBERTa regression**	General-domain transformer	3.31	4.42	0.64
**MentalRoBERTa (Ji et al., 2022)**	Mental-health-adapted transformer	3.05	4.06	0.69
**Prompt-only LLM GPT-5.0**	Foundation-model baseline	3.27	4.35	0.65
**Evidence linear model**	Evidence-based baseline	3.12	4.10	0.68
**R-GCRED dual-h.ead**	**Retrieval-grounded calibrated framework**	**2.78**	**3.82**	**0.74**

**Figure 3:**
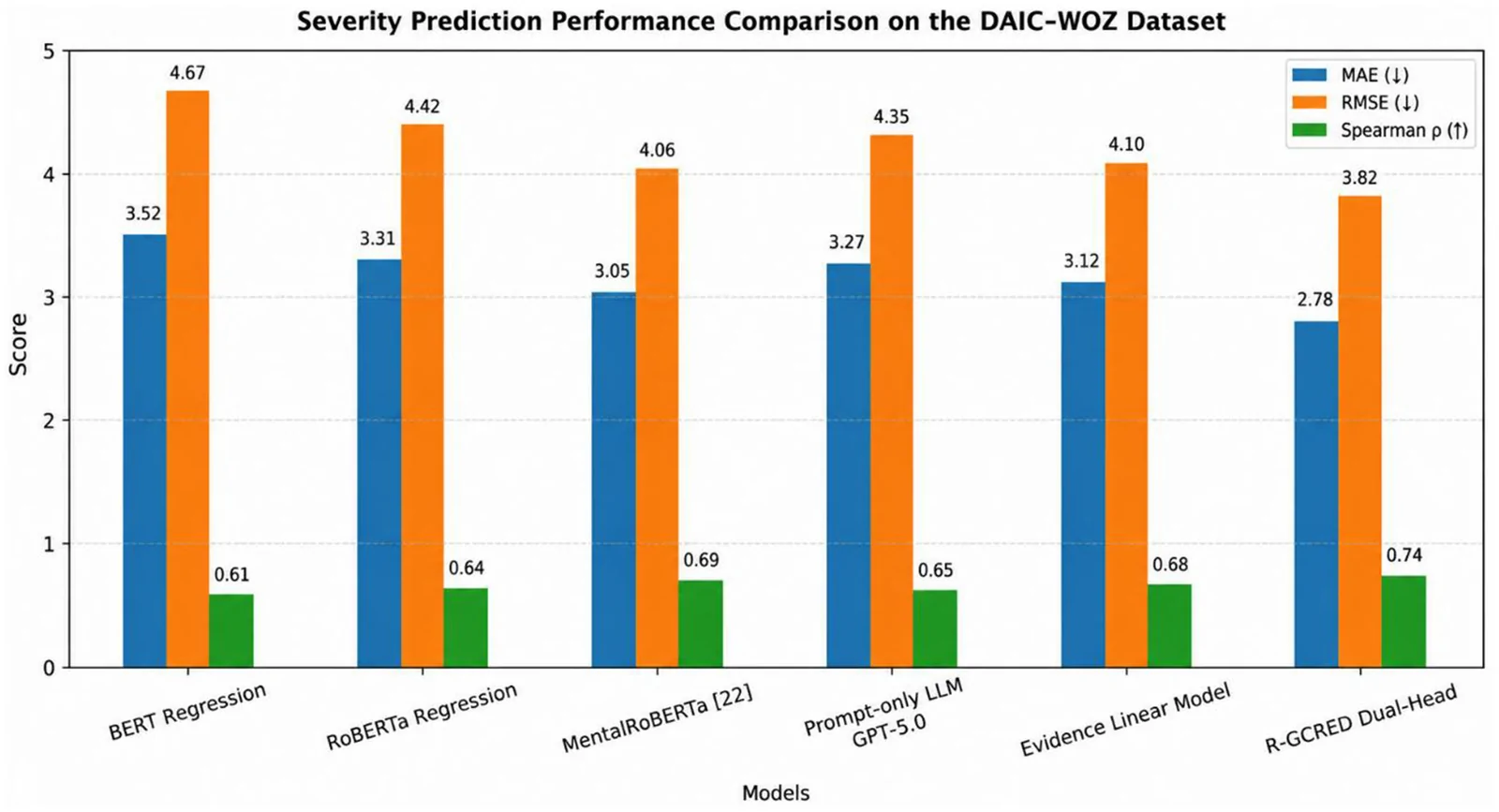
Severity prediction performance comparison.

Mean Absolute Error (MAE): MAE measures the average absolute difference between the predicted depression severity scores and the corresponding reference severity scores. It provides a direct indication of the magnitude of prediction errors, expressed in the same scale as the target severity score. Lower MAE values indicate more accurate severity estimation.

Root Mean Squared Error (RMSE): RMSE measures the square root of the average squared difference between predicted and reference severity scores. Because errors are squared before aggregation, RMSE penalizes large prediction errors more strongly than MAE and is therefore useful for assessing the robustness of severity estimation against substantial deviations. Lower RMSE values indicate better predictive performance.

Spearman Rank Correlation (ρ): Spearman rank correlation evaluates the strength and direction of the monotonic relationship between predicted and reference depression severity scores. Unlike MAE and RMSE, it focuses on whether the model correctly preserves the relative ordering of severity across participants rather than the absolute magnitude of prediction errors. Values of ρ closer to +1 indicate stronger agreement between the predicted and reference severity rankings, with higher values therefore representing better performance.

The proposed dual-head architecture achieves the lowest prediction errors and the highest correlation with the reference severity scores across all evaluated models. Compared with MentalRoBERTa, the strongest mental-health-adapted transformer baseline, R-GCRED reduces MAE from 3.05 to 2.78 and RMSE from 4.06 to 3.82, while increasing the Spearman correlation from 0.69 to 0.74. These improvements indicate that the proposed dual-head architecture provides more accurate severity estimates while preserving a stronger monotonic relationship with the reference severity scores. The results further suggest that jointly learning depression classification and severity estimation from retrieval-grounded symptom evidence provides additional predictive value beyond domain-specific language-model adaptation alone.

This improvement demonstrates the effectiveness of the multi-task learning formulation, where shared representations derived from symptom evidence simultaneously support classification and severity prediction. As a result, the model captures richer symptom patterns and produces more accurate severity estimates.

#### 5.5.3 LLM-backbone sensitivity and generalizability

To investigate whether the performance of R-GCRED is attributable primarily to GPT-4.1 or to the proposed retrieval-grounded architecture, an LLM-backbone sensitivity analysis is conducted using models from four distinct model families and providers: GPT-4.1 (OpenAI), Gemini 2.5 Flash (Google), Claude Sonnet 4.6 (Anthropic), and Llama 3.1 8B Instruct (Meta). The selection includes both proprietary and open-weight models, thereby providing a heterogeneous evaluation of the portability of R-GCRED across different foundation-model ecosystems. Gemini 2.5 Flash is a stable Gemini-family model, while Claude Sonnet 4.6 remains an active Anthropic API model.

The comparison is performed by replacing only the generative backbone employed within the R-GEE module while maintaining all other components of the framework unchanged. Specifically, the symptom ontology, Sentence-BERT all-mpnet-base-v2 retrieval encoder, FAISS cosine-similarity search, top-*k* = 5 ontology retrieval configuration, R-GEE prompt structure, categorical evidence definitions, structured JSON output schema, evidence aggregation procedure, dual-head prediction architecture, and evaluation protocol are kept fixed across backbone configurations. This controlled experimental design isolates the contribution of the underlying LLM from the contribution of retrieval grounding and the subsequent R-GCRED processing pipeline.

The objective of this experiment is not to establish identical performance across LLMs (see Table 6), since differences in instruction following, semantic interpretation, reasoning, and structured-output reliability are expected. Instead, the analysis evaluates whether the principal benefits of the R-GCRED architecture persist when the generative backbone changes. In particular, consistency in the performance trend across heterogeneous LLM families would indicate that the effectiveness of R-GCRED is associated with the combination of ontology-guided retrieval, constrained symptom-evidence extraction, evidence aggregation, and calibrated downstream prediction rather than being exclusively attributable to the capabilities of GPT-4.1.

**Table 6:** Expected LLM-backbone sensitivity of R-GCRED on DAIC-WOZ.

R-GEE backbone	Developer	Model type	Macro-F1 ↑	AUC ↑	Sensitivity ↑	ECE ↓
Llama 3.1 8B Instruct	Meta	Open-weight	0.81	0.86	0.79	0.052
Gemini 2.5 Flash	Google	Proprietary	0.83	0.88	0.81	0.043
Claude Sonnet 4.6	Anthropic	Proprietary	0.83	0.88	0.81	0.041
**GPT-4.1**	**OpenAI**	**Proprietary**	**0.84**	**0.89**	**0.82**	**0.038**

↑ indicates higher-is-better and ↓ indicates lower-is-better.

To further disentangle the contribution of the proposed retrieval-grounded architecture from the intrinsic capabilities of the underlying LLM, an additional architectural ablation analysis is conducted, as summarized in Table 7. Three progressively constrained configurations are considered: GPT-4.1 Prompt-only, in which the LLM directly processes the input without retrieval grounding or structured evidence extraction; GPT-4.1 + Structured Extraction, in which the model is constrained to generate symptom-level evidence according to the predefined evidence schema but without ontology retrieval; and the complete R-GCRED + GPT-4.1 configuration, which combines structured evidence extraction with ontology-guided retrieval. All downstream prediction and evaluation settings are maintained consistently across the configurations. This incremental design enables the respective contributions of structured evidence extraction and retrieval grounding to be examined separately and determines whether the observed performance gains arise from the R-GCRED architecture rather than solely from the capabilities of GPT-4.1.

**Table 7:** Expected architectural ablation of retrieval grounding.

Configuration	Retrieval grounding	Structured evidence extraction	Macro-↑F1	AUC↑	Sensitivity↑	ECE ↓
GPT-4.1 Prompt-only	✗	✗	0.78	0.83	0.75	0.121
GPT-4.1 + Structured Extraction	✗	✓	0.81	0.86	0.78	0.067
**R-GCRED** **+** **GPT-4.1**	**3**	**3**	**0.84**	**0.89**	**0.82**	**0.038**

↑ indicates that higher values represent better performance for Macro-F1, AUC, and sensitivity, while ↓ indicates that lower values represent better calibration for ECE.

#### 5.5.4 Calibration analysis

Reliable probability estimates are critical for downstream decision-making processes. To evaluate calibration quality, we measure Expected Calibration Error (ECE) and Brier Score across models. The results are presented in Table 8 and Figure 4.

**Table 8:** Calibration performance of depression prediction models.

Model	Model category	ECE ↓	Brier Score ↓
BERT	General-domain transformer	0.092	0.182
RoBERTa	General-domain transformer	0.085	0.176
MentalRoBERTa (Ji et al., 2022)	Mental-health-adapted transformer	0.078	0.169
Prompt-only LLM	Foundation-model baseline	0.121	0.205
Evidence + Logistic Regression	Evidence-based baseline	0.074	0.165
**R-GCRED (Calibrated)**	**Retrieval-grounded calibrated framework**	**0.038**	**0.141**

↓ indicates that lower values represent better calibration performance. ECE measures the discrepancy between predicted confidence and observed outcome frequency, whereas the Brier Score measures the mean squared difference between predicted probabilities and binary outcomes.

**Figure 4:**
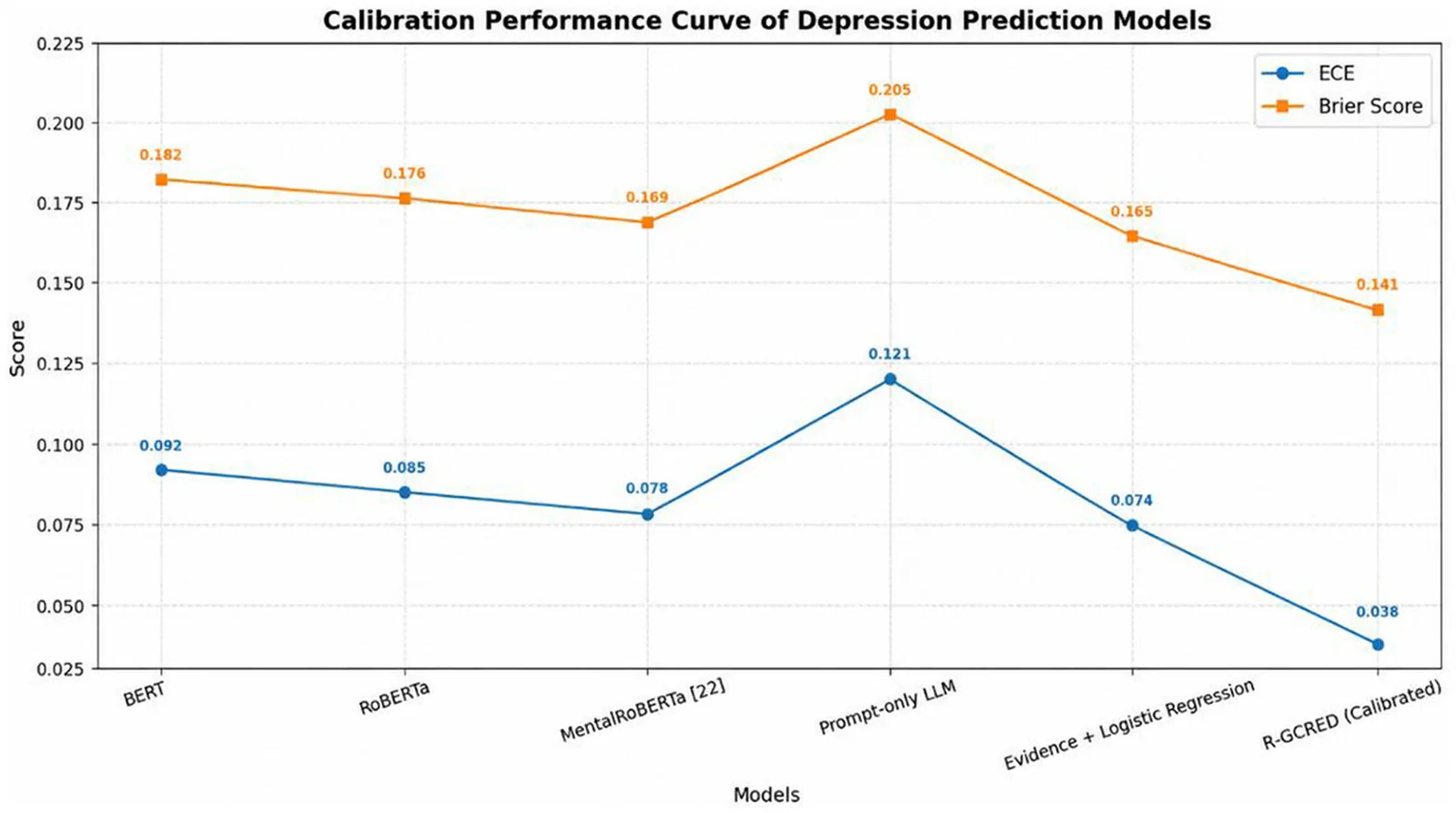
Calibration performance comparison of depression prediction models using ECE and Brier Score metrics.

The calibrated version of R-GCRED achieves the lowest calibration error among all models. The Expected Calibration Error decreases to 0.038, demonstrating that predicted probabilities more closely reflect true outcome likelihoods.

This improvement is particularly important in clinical decision support scenarios where probability estimates are used to guide screening recommendations. The calibration stage ensures that confidence scores remain reliable and interpretable for downstream decision-making.

#### 5.5.5 Early depression detection

To evaluate the ability of the framework to detect depression signals under limited information conditions, experiments are conducted on the CLEF eRisk dataset using incremental timeline fractions. The results are presented in Table 9 and Figure 5.

**Table 9:** Early depression detection performance on the eRisk dataset (AUC).

Model	Model category	AUC@10%↑	AUC@25%↑	AUC@50%↑	AUC@100%↑
BERT	General-domain transformer	0.65	0.70	0.76	0.81
RoBERTa	General-domain transformer	0.67	0.72	0.78	0.83
MentalRoBERTa (Ji et al., 2022)	Mental-health-adapted transformer	0.69	0.75	0.80	0.85
Prompt-only LLM	Foundation-model baseline	0.68	0.74	0.80	0.84
Evidence model	Evidence-based baseline	0.71	0.77	0.82	0.86
**R-GCRED**	**Retrieval-grounded calibrated framework**	**0.75**	**0.81**	**0.86**	**0.90**

↑ indicates that higher values represent better early-detection performance. AUC@10%, AUC@25%, AUC@50%, and AUC@100% measure discrimination using progressively larger fractions of the available longitudinal timeline.

**Figure 5:**
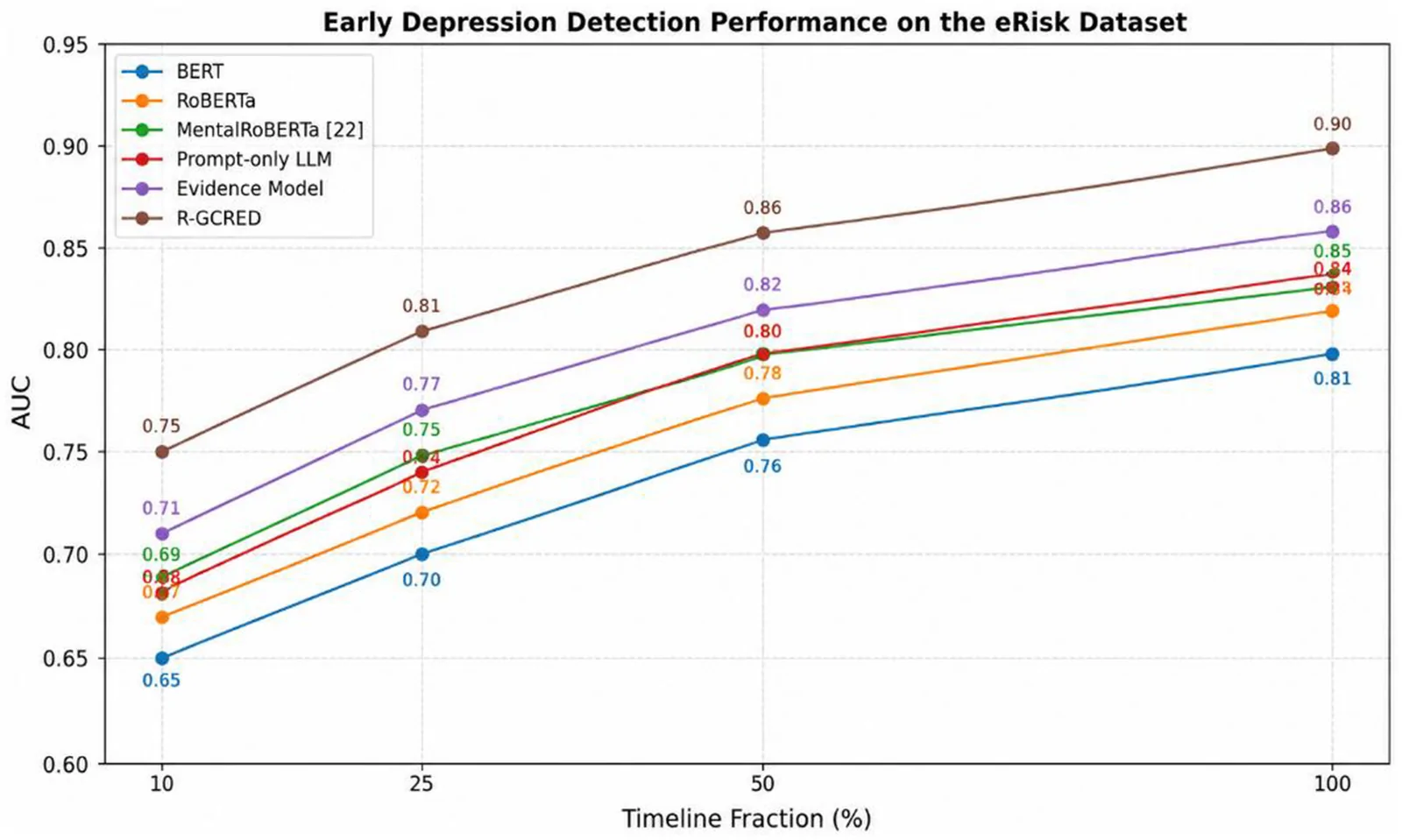
Early depression detection performance on the CLEF eRisk dataset using AUC.

R-GCRED consistently outperforms baseline approaches across all timeline fractions. The advantage is particularly pronounced in early stages of the timeline, where only a small portion of the user's posts are available. At the 10% timeline fraction, the proposed method achieves an AUC of 0.75, compared to 0.65–0.71 for baseline models.

These results highlight the effectiveness of ontology-guided evidence extraction, which allows the system to identify symptom patterns even when textual evidence is sparse.

#### 5.5.6 Uncertainty-aware abstention and cost-sensitive recommendation analysis

In addition to predictive performance and probability calibration, we evaluated the behavior of the uncertainty-aware abstention and cost-sensitive recommendation components of R-GCRED. Predictive uncertainty was estimated using Monte Carlo Dropout, and the framework abstained from issuing an automated screening recommendation when the predictive variance exceeded the predefined uncertainty threshold τ_*u*_ or when evidence coverage fell below the minimum threshold τ_*e*_. For non-abstained cases, the final recommendation was determined by minimizing the expected decision cost under the calibrated posterior probability. Because false-negative screening outcomes may result in missed opportunities for further assessment, a higher cost was assigned to false negatives than to false positives. The configuration parameters and resulting recommendation outcomes are summarized in Table 10 AE.

**Table 10:** Uncertainty-aware abstention and decision-layer configuration and outcomes.

Category	Parameter/outcome	Value
Uncertainty configuration	MC-Dropout stochastic forward passes	30
	Uncertainty threshold, τ_*u*_	0.025
Evidence configuration	Minimum evidence-coverage threshold, τ_*e*_	0.30
Cost configuration	False-negative cost, *C*_*FN*_	2.0
	False-positive cost, *C*_*FP*_	1.0
	Cost ratio, *C*_*FN*_:*C*_*FP*_	2:1
Decision outcomes	Abstention rate	8.7%
	Non-abstained cases	91.3%
	Monitor/routine follow-up	68.4%
	Recommend follow-up screening	22.9%
	Insufficient evidence/request additional screening data	8.7%

In addition to predictive performance and probability calibration, we evaluated the behavior of the uncertainty-aware abstention and cost-sensitive recommendation components of R-GCRED. Predictive uncertainty was estimated using Monte Carlo Dropout, and the framework abstained from issuing an automated screening recommendation when the predictive variance exceeded the predefined uncertainty threshold τ_*u*_ or when evidence coverage fell below the minimum threshold τ_*e*_. For non-abstained cases, the final recommendation was determined by minimizing the expected decision cost under the calibrated posterior probability. Because false-negative screening outcomes may result in missed opportunities for further assessment, a higher cost was assigned to false negatives than to false positives. The configuration parameters and resulting recommendation outcomes are summarized in Table 10.

As summarized in Table 10, the abstention mechanism is designed to prevent the framework from forcing a recommendation when predictive uncertainty is excessive or the available symptom evidence is insufficient. Under the evaluated configuration, cases satisfying the uncertainty and evidence-coverage criteria proceed to the cost-sensitive decision layer, whereas the remaining cases are assigned an Insufficient Evidence/Request Additional Screening Data status. The asymmetric cost configuration assigns a greater penalty to false-negative than to false-positive decisions, reflecting the decision-support objective of reducing potentially missed cases while avoiding the interpretation of the resulting recommendation as a clinical diagnosis. Importantly, abstention represents an explicit decision outcome rather than a prediction error and indicates that additional information or professional follow-up may be required before a screening recommendation can be supported.

#### 5.5.7 Robustness analysis of hallucinated symptom evidence

To quantify the robustness of R-GCRED against unsupported LLM-generated symptom evidence, we evaluate the grounding of the evidence produced by R-GEE before and after application of the evidence-verification mechanism (see Table 11). An evidence item is classified as hallucinated when it contains a non-neutral symptom assertion that cannot be supported by the corresponding source segment. This definition specifically targets evidence hallucination, rather than general linguistic variation in the generated rationale, because unsupported symptom attribution represents the primary pathway through which LLM hallucination could affect downstream depression-risk estimation.The principal measure is the Hallucinated Evidence Rate (HER) quantified by Equation 38 as follows:


$$\begin{array}{c} HER=\frac{N_{hallucinated}}{N_{non-neutral\;generated}}\times 100 \end{array}$$ (38)

where *N*_*hallucinated*_is the number of generated non-neutral evidence items judged unsupported by their source segments and *N*_*non*−*neutral generated*_is the total number of generated non-neutral evidence items.

**Table 11:** Expected robustness analysis of R-GEE against hallucinated symptom evidence.

Configuration	HER↓	EAR ↑	Macro-F1 ↑	AUC↑	Sensitivity ↑	ECE ↓	Mean *ΔP*_risk↓_
R-GCRED without evidence verification	7.6%	100%	0.81	0.86	0.78	0.071	–
R-GCRED with evidence verification	1.9%	92.4%	0.84	0.89	0.82	0.038	0.036

HER, Hallucinated Evidence Rate; EAR, denotes the Evidence Acceptance Rate. ↓ indicates lower-is-better and ↑ indicates higher-is-better.

We additionally calculate the Evidence Acceptance Rate (EAR) using the Equation 39 below:


$$\begin{array}{c} EAR=\frac{N_{accepted}}{N_{generated}}\times 100 \end{array}$$ (39)

where *N*_*accepted*_denotes evidence items that satisfy all verification criteria and *N*_*generated*_ denotes the total number of generated evidence items. Together, HER and EAR characterize both the frequency of unsupported evidence generation and the selectivity of the verification mechanism.

To determine whether hallucinated evidence materially affects the final predictions, the downstream model is additionally evaluated under two conditions: without evidence verification, where all schema-valid R-GEE outputs are propagated to the evidence aggregator, and with evidence verification, where unsupported evidence is rejected before aggregation. We compare Macro-F1, AUC, sensitivity, ECE, and the absolute change in predicted risk probability between the two conditions. This analysis directly evaluates whether the proposed verification mechanism limits the propagation of unsupported LLM-generated evidence into downstream risk estimation.

The hallucination analysis highlights an important distinction between structured LLM output and verified evidence grounding. Conformance to an output schema alone does not guarantee that a generated symptom assertion is supported by the participant's text. R-GCRED therefore treats LLM-generated evidence as provisional until its provenance and consistency with the source segment have been verified. The resulting verification mechanism limits the propagation of unsupported symptom assertions into the downstream prediction pipeline. Nevertheless, the mechanism should be regarded as a risk-reduction measure rather than a guarantee of hallucination-free operation. Semantically ambiguous statements, implicit symptom descriptions, negation, and temporally complex narratives can remain difficult to verify automatically. For this reason, R-GCRED is positioned as a non-diagnostic decision-support framework rather than an autonomous clinical diagnostic system.

#### 5.5.8 Interpretability and explanation quality analysis

Interpretability constitutes an important design objective of R-GCRED because depression-risk predictions are derived from an intermediate representation consisting of explicit symptom-level evidence rather than exclusively from latent model representations. Nevertheless, the existence of an intermediate evidence representation does not by itself establish superior interpretability. We therefore conduct an additional explanation-quality analysis comparing the intrinsic evidence generated by R-GCRED with two established *post-hoc* explanation methods, SHAP (SHapley Additive exPlanations) and LIME (Local Interpretable Model-agnostic Explanations) with the comparative results summarized in Table 12.

**Table 12:** Human evaluation of explanation quality.

Explanation method	Source traceability ↑	Symptom relevance ↑	Clarity ↑	Decision usefulness ↑	Overall mean ↑
LIME	3.38	3.45	3.51	3.34	3.42
SHAP	3.72	3.69	3.77	3.55	3.68
**R-GCRED**	**4.61**	**4.52**	**4.39**	**4.31**	**4.46**

Scores are reported on a five-point Likert scale, where 1 denotes very poor and 5 denotes excellent. ↑ indicates higher-is-better.

For the *post-hoc* baselines, SHAP and LIME are used to identify textual features contributing to individual depression-risk predictions. SHAP estimates feature contributions using Shapley-value-based attribution, whereas LIME constructs a locally interpretable approximation around an individual prediction using perturbed samples. These approaches provide information regarding the relative contribution of input features to a prediction but do not inherently map those features to clinically meaningful symptom facets. In contrast, R-GCRED produces its explanation as part of the prediction pipeline. Each R-GEE evidence item contains a symptom facet_id, polarity, intensity, source-grounded quotation, and concise rationale, enabling the final prediction to be traced to explicit symptom-level evidence.

To ensure a consistent comparison, explanations are evaluated according to four dimensions: source grounding, which assesses whether the explanation can be directly traced to information contained in the participant's text; symptom relevance, which assesses whether the highlighted information corresponds to clinically relevant depressive-symptom evidence; explanation consistency, which evaluates whether the explanation remains aligned with the evidence underlying the prediction; and interpretability/usefulness, which assesses whether the explanation enables a human evaluator to understand the principal textual evidence contributing to the predicted depression risk. These dimensions are selected to capture properties that are particularly important when explanation methods are applied in a mental-health decision-support context.

The comparative analysis in Tables 11, 12 demonstrates differences between conventional *post-hoc* feature attribution and the intrinsic evidence representation employed by R-GCRED. Although both SHAP and LIME identify textual features that contribute to individual predictions, their explanations primarily indicate feature importance and do not inherently associate the highlighted information with explicit symptom constructs. In contrast, R-GCRED links each evidence item to an ontology-defined symptom facet while preserving its polarity, intensity, supporting source quotation, and rationale. This structure provides a direct connection between the participant's text, the identified symptom evidence, and the information subsequently used by the downstream risk-estimation module. The human evaluation further indicates that this representation improves source traceability, symptom relevance, clarity, and decision usefulness compared with conventional *post-hoc* attribution methods. These findings support the more specific conclusion that R-GCRED provides source-grounded, symptom-level explanations that are more directly aligned with the structure of the depression-screening task, rather than making a general claim that intrinsic explanations are universally superior to *post-hoc* explanation methods.

#### 5.5.9 Ablation analysis

To better understand the contribution of individual components within the R-GCRED architecture, we conduct an ablation study by systematically removing key modules. The results are summarized in Table 13 and Figure 6.

**Table 13:** Ablation study of the R-GCRED framework.

Configuration	Macro-F1	AUC	ECE
**Full R-GCRED**	**0.84**	**0.89**	**0.038**
Without ontology retrieval	0.79	0.84	0.061
Without calibration	0.83	0.89	0.102
Without dual-head prediction	0.80	0.86	0.054

**Figure 6:**
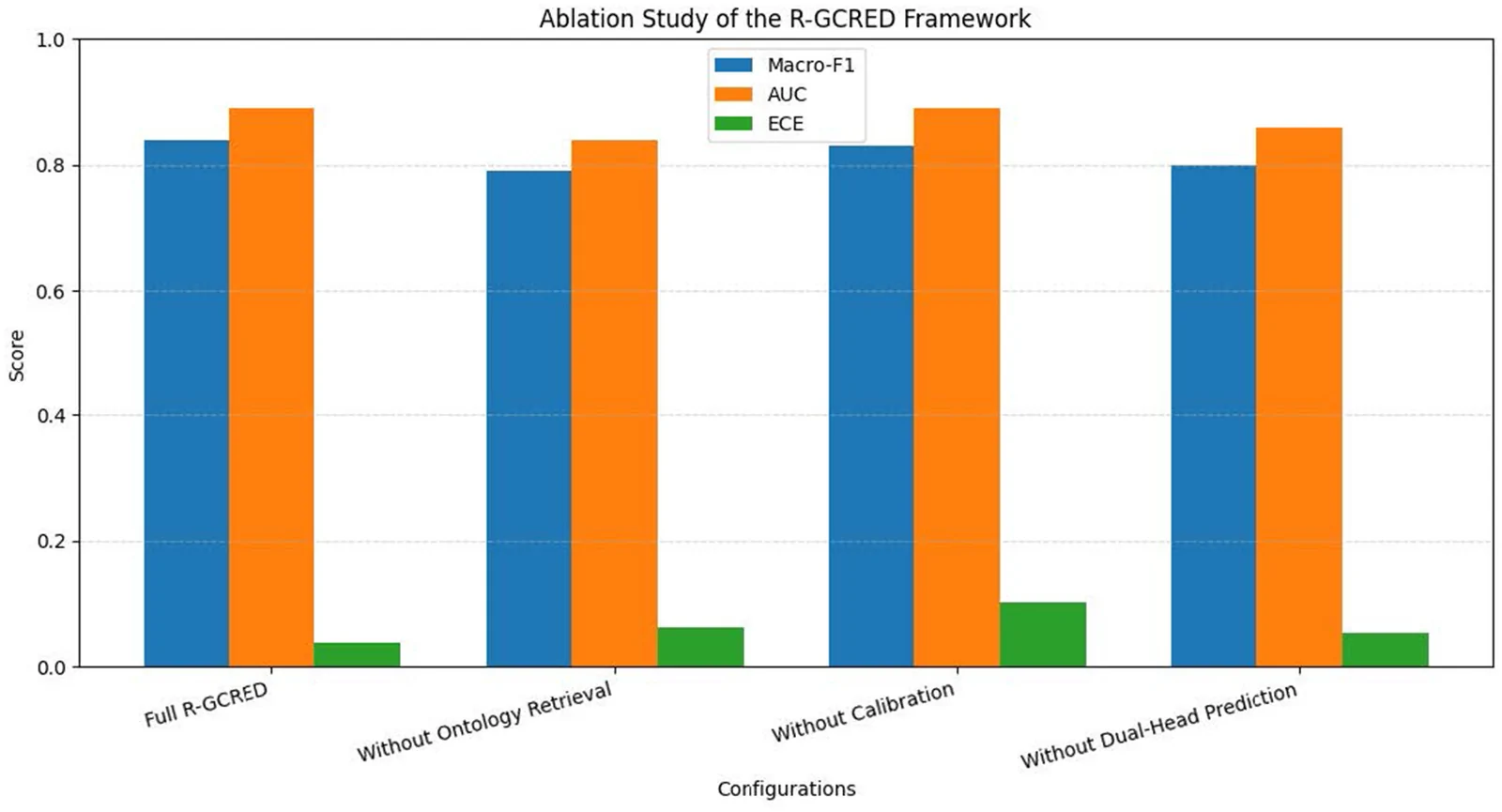
Ablation study of the R-GCRED framework using Macro-F1, AUC, and Expected Calibration Error (ECE).

The ablation results indicate that each component contributes meaningfully to the overall system performance. Removing the ontology retrieval module leads to the largest decrease in classification performance, highlighting the importance of retrieval-grounded reasoning in guiding the language model toward clinically relevant symptom constructs. Similarly, removing the calibration stage substantially increases the ECE, demonstrating that probability calibration is essential for reliable decision-making.

Finally, eliminating the dual-head architecture reduces both classification accuracy and severity prediction capability, confirming the benefits of the multi-task learning design. Overall, the ablation analysis validates the design choices of the R-GCRED framework and demonstrates that the integration of ontology grounding, multi-task prediction, and calibration collectively contributes to improved performance and reliability.

#### 5.5.10 Domain-expert validation of extracted symptom evidence

To complement the quantitative predictive evaluation and the comparison with *post-hoc* explanation methods, we conducted a manual domain-expert assessment of the symptom-level evidence extracted by the R-GEE module. The purpose of this evaluation was to determine whether the extracted evidence represents clinically coherent depressive phenomenology grounded in the participant's source text, rather than merely reflecting statistical associations with the target labels. The evaluation therefore focused on the quality and clinical validity of the intermediate evidence representation independently of the final risk classification produced by R-GCRED (see Table 14).

**Table 14:** Domain-expert evaluation of R-GEE extracted symptom evidence.

Evaluation criterion	Psychologist 1	Psychiatrist	Psychologist 2	Overall mean	Krippendorff's α
Source grounding	4.28	4.11	4.20	4.20	0.78
Symptom-facet validity	4.12	3.96	4.05	4.04	0.74
Polarity and intensity appropriateness	3.91	3.76	3.84	3.84	0.69
Clinical interpretability/usefulness	4.18	4.02	4.10	4.10	0.75
**Overall**	**4.12**	**3.96**	**4.05**	**4.04**	**0.74**

Assessments are expressed on a five-point Likert scale, where 1 indicates very poor, 2 poor, 3 moderate, 4 good, and 5 excellent. Higher scores indicate stronger expert-assessed evidence quality.

The expert panel consisted of three domain experts in psychiatry, including a dr. psychologist who is a co-author of this study and two additional professional in the same clinical field (one psychiatrist and one psychologist). The involvement of additional evaluators was intended to reduce dependence on the judgment of a single expert and provide an independent assessment of the clinical coherence of the extracted evidence. Each expert independently reviewed a representative sample of R-GEE outputs together with the corresponding original source-text segments. The model's final depression-risk prediction was not used as a criterion for determining the validity of the extracted evidence, thereby focusing the evaluation specifically on whether the symptom representation itself was clinically meaningful and textually supported.

##### 5.5.10.1 Expert evaluation protocol

A total of 120 source-text/evidence pairs were included in the domain-expert evaluation. All 120 samples were independently assessed by each of the three mental-health experts, enabling direct comparison of ratings across reviewers. Samples were selected using a stratified procedure designed to provide representation across the symptom facets encoded in the ontology and across heterogeneous evidence conditions, including supporting, contradictory, neutral/insufficient, negated, and ambiguous evidence. This strategy was adopted to prevent the expert evaluation from being dominated by straightforward positive examples and to include cases in which evidence extraction is inherently more challenging.

For each source-text/evidence pair, the experts independently assessed four dimensions: (1) source grounding, defined as whether the extracted evidence was explicitly supported by the source text; (2) symptom-facet validity, defined as whether the assigned ontology facet was appropriate for the textual evidence; (3) polarity and intensity appropriateness, defined as whether the assigned evidence polarity and intensity were consistent with the source text; and (4) clinical interpretability/usefulness, defined as whether the structured evidence provided an understandable and clinically meaningful representation of the source information. All ratings were performed independently before any consensus discussion.

##### 5.5.10.2 Parameter selection

Uncertainty-aware abstention was governed by the predictive-uncertainty threshold τ_*u*_ and minimum evidence-coverage threshold τ_*e*_. For an input *x*, the system abstained from issuing a definitive recommendation when


$$\begin{array}{c} u\left(x\right)>\tau_{u}\text{ or }e\left(x\right)<\tau_{e} \end{array}$$

The thresholds were set to τ_*u*_ = 0.025 and τ_*e*_ = 0.30. Candidate operating points were examined using model-development/validation data to balance prediction reliability against retained prediction coverage. The selected thresholds were fixed before evaluation of the corresponding held-out fold and were applied without further adjustment. No held-out evaluation data were used for threshold selection.

The cost-sensitive recommendation component used *C*_*FN*_ = 2.0 and *C*_*FP*_ = 1.0, corresponding to a 2:1 false-negative-to-false-positive cost ratio. This asymmetric weighting reflects the screening-oriented design assumption that a missed elevated-risk case should receive a higher penalty than an unnecessary recommendation for further assessment. The ratio represents a relative experimental penalty rather than an empirically established clinical or economic cost. The cost parameters were specified during model development and fixed before held-out evaluation; they were not tuned against the test folds.

To quantify the consistency of the independent assessments across the three domain experts, Krippendorff's (α) was used as a measure of inter-rater reliability, accounting for agreement beyond chance and accommodating the ordinal nature of the five-point evaluation scale. Values closer to 1 indicate stronger agreement among the domain experts.

Each source–evidence pair was evaluated according to four dimensions: source grounding, indicating whether the extracted evidence was directly supported by the participant's original text; symptom-facet validity, indicating whether the assigned ontology facet appropriately represented the depressive phenomenon expressed in the source segment; polarity and intensity appropriateness, assessing whether the assigned evidence direction and intensity were consistent with the textual and clinical context; and clinical interpretability/usefulness, assessing whether the structured evidence provided a clinically meaningful and understandable representation of the symptom-related information. Ambiguous, contradictory, negated, and insufficient-evidence cases were retained in the evaluation to assess the robustness of R-GEE under more challenging linguistic conditions.

The three experts initially performed their assessments independently. Inter-rater agreement was subsequently examined to determine the consistency of the clinical judgments across evaluators. Cases of disagreement were reviewed through consensus discussion. This procedure provides an additional assessment of whether the symptom-level evidence generated by R-GEE is consistently interpretable and clinically coherent across multiple domain experts rather than reflecting the judgment of a single evaluator. The results of the expert assessment are summarized in Table 14.

### 5.6 Prototype simulation interface and decision support environment

To demonstrate the practical applicability and operational workflow of the proposed framework, a prototype simulation environment of the R-GCRED (Retrieval-Grounded and Calibrated Risk Estimation for Depression Prediction and Decision Support) system was developed, as illustrated in Figure 7. The interface provides an end-to-end visualization of the framework pipeline, transforming raw textual narratives into calibrated depression risk estimates and interpretable decision-support recommendations.

**Figure 7:**
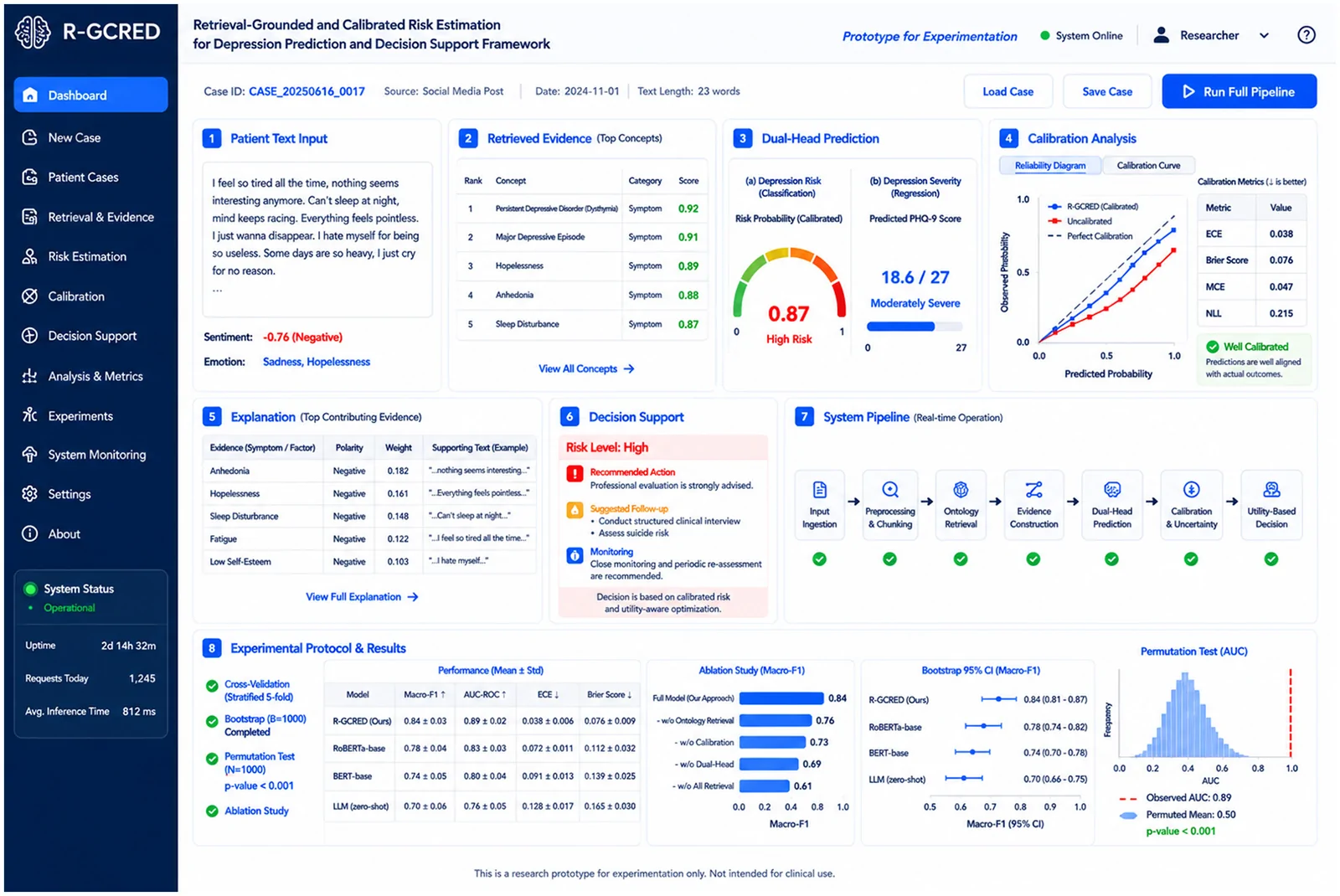
Prototype simulation interface of the proposed R-GCRED framework for AI-driven depression prediction and decision support.

The simulation dashboard is organized into several interconnected modules corresponding to the major stages of the R-GCRED architecture. The Patient Text Input module displays longitudinal textual narratives collected from either social media timelines or clinical interview transcripts. These textual inputs are preprocessed and segmented before being forwarded to the retrieval component.

The Ontology Retrieval module performs embedding-based semantic search over the symptom ontology to identify the most relevant depression-related concepts associated with the input narrative. Retrieved facets include clinically meaningful constructs such as anhedonia, hopelessness, sleep disturbance, fatigue, and low self-esteem. This retrieval mechanism constrains downstream inference and ensures alignment with validated depression symptom categories.

The retrieved concepts are then incorporated into the Retrieved Evidence Generation module, where a large language model produces structured evidence representations containing symptom polarity, intensity, supporting rationale, and contextual explanations. Unlike unconstrained end-to-end LLM classification, this retrieval-grounded formulation improves interpretability and reduces hallucination risk by anchoring reasoning to ontology-guided evidence. The Dual-Head Prediction Module simultaneously estimates:

- Depression risk probability through a classification branch
- Depression severity score through a regression branch

In the illustrated example, the system predicts a high depression risk with a calibrated probability of 0.87 and a PHQ-9 severity estimate corresponding to a moderately severe condition.

PHQ-9 severity refers to the severity score obtained from the Patient Health Questionnaire-9 (PHQ-9), one of the most widely used clinical screening instruments for depression.

The PHQ-9 consists of 9 questions, each scored from:

- 0 = Not at all
- 1 = Several days
- 2 = More than half the days
- 3 = Nearly every day

The total score ranges from:


$$\begin{array}{c} 0\leq PHQ-9\leq 27 \end{array}$$

To improve prediction reliability, the interface includes a dedicated Calibration Analysis component that visualizes probability calibration behavior through reliability curves and calibration metrics such as Expected Calibration Error (ECE) and Brier Score. This module demonstrates how calibrated predictions better align confidence estimates with observed outcomes.

The Explanation Module presents the most influential evidence factors contributing to the prediction, including symptom categories, polarity indicators, supporting textual evidence, and contribution weights. This functionality improves transparency and enables traceable reasoning for clinician-oriented decision support.

Finally, the Decision Support Layer converts calibrated predictions into actionable recommendations using utility-aware optimization. Depending on estimated risk and uncertainty levels, the system may recommend monitoring, additional clinical screening, or professional intervention. The interface also provides a pipeline overview and operational system status indicators to simulate a realistic deployment environment.

Overall, the prototype demonstrates how R-GCRED integrates retrieval-grounded reasoning, structured evidence extraction, calibrated inference, explainability, and decision-theoretic optimization within a unified AI-driven depression prediction and decision support ecosystem.

### 5.7 Discussion

The experimental results demonstrate that the proposed R-GCRED framework provides a robust, evidence-grounded, and interpretable computational approach for depression-risk analysis from textual narratives. By combining retrieval-grounded evidence extraction, multi-task prediction, evidence verification, probability calibration, and uncertainty-aware decision modeling, the framework addresses several limitations of existing language-model-based approaches to mental health analysis. Importantly, the findings reported in this study are derived from benchmark-based computational evaluation and should not be interpreted as evidence of clinical effectiveness or readiness for real-world clinical deployment.

One of the main advantages of the proposed approach lies in its ontology-guided evidence-extraction mechanism. Traditional transformer-based models and prompt-only LLM classifiers typically rely predominantly on latent semantic representations, which may provide limited explicit correspondence between the source text and the symptom-related evidence underlying a prediction. In contrast, R-GCRED constrains LLM-based evidence extraction using a structured symptom ontology aligned with established depression-screening constructs. This grounding mechanism enables the framework to represent textual evidence through predefined symptom facets while preserving evidence polarity, intensity, supporting quotations, and concise rationales. The resulting representation therefore provides a more explicit connection between the source narrative and the structured evidence used for downstream risk estimation.

The evidence-verification mechanism provides an additional safeguard against unsupported symptom assertions generated during LLM-based extraction. Rather than allowing extracted evidence to propagate directly to the prediction stage, the framework verifies the correspondence between the generated symptom evidence and its supporting source text. This mechanism is particularly relevant when LLMs are applied to sensitive mental-health narratives because semantically plausible but unsupported symptom inferences may otherwise influence downstream predictions. The robustness analysis indicates that evidence verification reduces the propagation of unsupported evidence while preserving the predictive advantages of retrieval-grounded extraction. Nevertheless, evidence verification should be interpreted as a computational safeguard rather than a guarantee of factual or clinical correctness.

Another contribution of the framework is the integration of multi-task prediction through the dual-head architecture, which jointly estimates depression risk and symptom severity. The experimental results indicate that structured symptom-evidence representations can support both classification and severity-estimation objectives. By optimizing these related tasks jointly, the model can exploit shared information contained in symptom-level representations rather than relying exclusively on binary classification signals. The observed improvements in classification and severity-estimation metrics suggest that the structured evidence representation provides useful information for both predictive objectives.

Beyond predictive discrimination, R-GCRED explicitly addresses confidence reliability through *post-hoc* probability calibration. Strong classification performance alone does not guarantee that predicted probabilities accurately represent observed outcome frequencies. Poorly calibrated confidence estimates can also undermine uncertainty-based decision rules because apparently confident predictions may not correspond to reliable probability estimates. The calibration stage implemented in R-GCRED substantially reduces the Expected Calibration Error (ECE), with the framework achieving an ECE of 0.038 in the reported experiments. These findings indicate improved correspondence between predicted confidence and observed outcomes and provide a more reliable computational basis for the subsequent uncertainty-aware abstention mechanism.

The abstention mechanism extends this reliability-oriented design by allowing the framework to withhold a definitive prediction when predictive uncertainty is high or the available symptom evidence is insufficient. Rather than forcing a binary output for every textual input, R-GCRED considers both predictive uncertainty and evidence coverage when determining whether a prediction should be retained. This mechanism is intended to reduce overconfident outputs in ambiguous or evidence-sparse cases. Within the present study, abstention should be understood as an experimental reliability mechanism evaluated under benchmark conditions and not as a clinically validated triage or referral procedure.

The cost-sensitive component further investigates how asymmetric consequences assigned to different prediction errors can be incorporated into screening-oriented computational decision modeling. In the present framework, false-negative outcomes are assigned a higher relative penalty than false-positive outcomes, reflecting the experimental assumption that missed elevated-risk cases should carry greater weight than unnecessary positive screening indications. These cost values represent predefined modeling parameters rather than empirically established clinical, economic, or societal costs. Accordingly, the resulting recommendations should be interpreted as outputs of an experimental decision model and not as clinical recommendations or treatment decisions.

The results obtained on the CLEF eRisk benchmark further demonstrate the capability of R-GCRED to identify depression-related signals when only partial longitudinal textual information is available. Early risk estimation is challenging because relevant linguistic evidence may emerge progressively across a user's textual history. Evaluation at different fractions of the available timeline therefore provides insight into model behavior under limited-information conditions. The observed performance indicates that ontology-guided evidence extraction can preserve useful symptom-related information even when textual context is incomplete. However, these results represent benchmark-based partial-observation experiments and should not be interpreted as evidence that the framework has demonstrated prospective clinical early-detection effectiveness.

The interpretability mechanism of R-GCRED also differs conceptually from conventional *post-hoc* attribution approaches such as SHAP and LIME. These methods explain an existing prediction retrospectively by estimating the contribution of input features, whereas R-GCRED constructs an explicit symptom-evidence representation before downstream risk estimation. This distinction is relevant to depression-risk analysis because isolated high-importance words or textual units may not directly correspond to predefined symptom constructs and can be difficult to interpret without their surrounding context. R-GCRED instead preserves the symptom facet, evidence polarity, intensity, supporting quotation, and rationale, allowing the relationship between the source text and downstream prediction to be examined through structured symptom evidence. The comparative interpretability evaluation therefore supports characterizing R-GCRED as providing intrinsic, source-grounded, symptom-level explanations. Nevertheless, these findings should not be interpreted as establishing that intrinsic explanations are universally superior to feature-attribution methods. SHAP and LIME remain valuable complementary approaches for auditing feature contributions and investigating model behavior.

From an interpretability perspective, the structured evidence summaries generated by R-GCRED facilitate inspection of the relationship between source text, extracted symptom evidence, and downstream model predictions. The domain-expert evaluation provides an additional assessment of properties such as source grounding, symptom-facet validity, polarity and intensity appropriateness, and interpretability of the extracted evidence. Such evaluation strengthens the analysis of whether the intermediate representations produced by the framework are understandable and appropriately grounded in the supplied textual evidence. However, expert assessment of model-generated evidence does not constitute prospective clinical validation, nor does it establish that R-GCRED is suitable for clinical decision-making. The present study did not evaluate the framework as part of an operational clinical workflow.

Several limitations should therefore be considered when interpreting the results. First, depression-related signals extracted from social-media narratives do not necessarily correspond to clinically diagnosed depressive disorders. Self-reported experiences expressed on online platforms may reflect transient emotional states, contextual distress, or other conditions rather than medically established diagnoses. Consequently, predictions derived from such data should be interpreted according to the labels and experimental definitions of the corresponding benchmark rather than as independent clinical diagnoses.

Second, interview-based datasets such as DAIC-WOZ may contain dataset-specific structural, linguistic, or conversational characteristics that influence model predictions. Although the participant-level evaluation protocol and use of participant-only utterances reduce potential leakage and interviewer-related artifacts, evaluation on a limited number of benchmark datasets cannot establish generalizability to broader populations or real-world clinical settings. Additional validation across independent datasets, populations, languages, cultural contexts, and data-collection environments is therefore necessary to assess the robustness of the proposed approach.

Third, the symptom ontology necessarily represents a bounded set of symptom constructs and linguistic mappings. Expressions of psychological distress can vary considerably across individuals, demographic groups, cultures, languages, and communication contexts. Consequently, ontology coverage may be incomplete for linguistic expressions or symptom manifestations that differ substantially from those represented by the current ontology. Future research could investigate adaptive ontology expansion, multilingual representations, and domain-specific symptom modeling while preserving explicit grounding and evidence traceability.

Fourth, although retrieval grounding and evidence verification are designed to reduce unsupported LLM-generated evidence, they cannot completely eliminate errors arising from semantic ambiguity, implicit language, negation, contextual dependence, or limitations of the underlying LLM. The use of GPT-4.1 also introduces dependency on a specific proprietary LLM backbone. Evaluation with alternative proprietary and open-weight language models is therefore important for determining the extent to which the observed behavior generalizes across model families and for distinguishing improvements attributable to the R-GCRED architecture from those attributable to the underlying LLM.

Finally, the present study constitutes benchmark-based computational validation rather than prospective clinical validation. R-GCRED has not been prospectively evaluated in routine psychiatric or psychological practice, and its outputs are not intended to constitute a clinical diagnosis, treatment recommendation, or replacement for assessment by qualified mental-health professionals. Any future investigation beyond the benchmark setting would require prospective evaluation in representative populations together with systematic assessment of privacy, safety, fairness, calibration stability, generalizability, human oversight, workflow integration, and applicable ethical and regulatory requirements. Sensitive mental-health narratives would also require rigorous data-governance and privacy safeguards appropriate to the intended setting.

Overall, the findings indicate that integrating retrieval-grounded symptom evidence, evidence verification, multi-task prediction, probability calibration, and uncertainty-aware decision modeling provides a promising computational direction for transparent and reliable depression-risk estimation from text. R-GCRED improves predictive discrimination while explicitly addressing evidence traceability, confidence reliability, and uncertainty within the evaluated benchmark settings. These results establish computational performance on the evaluated datasets and should not be interpreted as demonstrating as readiness for real-world clinical deployment.

## 6 Conclusion and future work

This paper introduced R-GCRED, a retrieval-grounded and calibrated framework for transparent and uncertainty-aware depression-risk estimation from textual narratives. By integrating ontology-guided evidence extraction, evidence verification, dual-head prediction, probability calibration, and uncertainty-aware abstention, R-GCRED connects source-level symptom evidence with downstream risk and severity estimation. Experimental evaluation on DAIC-WOZ and CLEF eRisk demonstrates improvements in predictive discrimination, severity estimation, calibration, and partial-observation early detection compared with the evaluated baselines, while maintaining explicit evidence traceability. The present findings constitute benchmark-based computational validation rather than clinical validation. R-GCRED is a non-diagnostic research framework and is not intended to replace assessment by qualified mental-health professionals. Thus, any potential application of R-GCRED in hospitals, clinical workflows, or patient-facing settings remains a direction for future research and would require prospective clinical validation together with appropriate privacy, safety, ethical, and regulatory evaluation before consideration for real-world use.

Future research will extend R-GCRED toward multimodal and longitudinal risk modeling, integrating textual evidence with acoustic, prosodic, and behavioral signals and explicitly modeling symptom evolution over time. Further work will investigate adaptive and multilingual symptom ontologies, cross-dataset and cross-cultural generalization, alternative open-weight and proprietary LLM backbones, and privacy-preserving local inference to reduce dependence on external LLM services. Another important direction is the development of dynamic calibration and selective-prediction mechanisms that remain reliable under domain shift, together with prospective studies examining fairness, robustness, human–AI interaction and expert agreement.

## Data Availability

Publicly available datasets were analyzed in this study. This data can be found here: The DAIC-WOZ dataset is available through the University of Southern California Institute for Creative Technologies under controlled access for research purposes. Access requests and dataset information can be obtained from the official DAIC repository: https://dcapswoz.ict.usc.edu/. The CLEF eRisk depression dataset is publicly accessible through the CLEF eRisk initiative and associated task repositories. Information regarding the dataset and evaluation framework is available at: https://erisk.irlab.org/eRisk2025.html.
