# Supplementary material for: R-GCRED: a retrieval-grounded and calibrated risk estimation for depression prediction and screening recommendation framework
Source: Front Artif Intell. 2026 Oct 6;9:1931579. doi: 10.3389/frai.2026.1931579 (PMC13680418; doi:10.3389/frai.2026.1931579)
Supplement: Supplementary file 1 [file Supplementary_file_1.docx]

**Supplementary Material:**

**S1. Prompt Template for Retrieval-Grounded Evidence Extraction (R-GEE)**

**S1.1 Purpose and Prompt Instantiation**

The Retrieval-Grounded Evidence Extraction (R-GEE) module uses a fixed prompt structure to transform each segmented textual unit into ontology-aligned structured evidence. For each input segment $s_{i}$, the retrieval module identifies the top-$k=5$ most semantically relevant symptom facets using all-mpnet-base-v2 embeddings and cosine-similarity search over the FAISS ontology index. The retrieved facet set $F_{i}$and the corresponding input segment $s_{i}$are then inserted into the prompt template below. The instructions, extraction criteria, categorical definitions, and output schema remain fixed across instances; only $s_{i}$and $F_{i}$are dynamically substituted. LLM inference is performed using GPT-4.1 through the OpenAI API with temperature = 0.0, top-p = 1.0, and a maximum output length of 512 tokens, consistent with the implementation described in Section 5.2.3.

These parameters come directly from the manuscript rather than being newly introduced.

**S1.2 System Instruction**

**System instruction**

You are the evidence-extraction component of a depression-screening decision-support framework. Your task is to identify and structure textual evidence relevant only to the depression symptom facets supplied in the retrieved ontology context.

You are not performing a clinical diagnosis and must not provide treatment advice, a depression probability, a final depression classification, or a screening recommendation.

Base every extracted evidence item strictly on the supplied input text and the retrieved symptom-facet definitions. Do not introduce symptoms, events, intentions, severity information, or clinical conclusions that are not supported by the input text. Do not assign a symptom facet that is not included in the retrieved facet set.

Return the result only according to the required structured JSON schema.

This preserves the manuscript's stated role for the LLM: it is used primarily for **grounded evidence structuring rather than end-to-end labeling**.

**S1.3 Retrieval Context and User Prompt Template**

**Retrieved ontology context**

The following symptom facets were retrieved as the top-$k$ ontology candidates for the current text segment:

{RETRIEVED_FACETS}

Each retrieved facet may contain:

- facet_id: unique ontology identifier;
- facet_name: symptom-facet name;
- definition: clinical/ontology description;
- disambiguation: information used to distinguish genuine symptom evidence from ambiguous expressions;
- examples: representative linguistic expressions associated with the facet.

**Input text segment**

{INPUT_SEGMENT}

**Task**

Evaluate the input segment with respect to the retrieved symptom facets only. For each retrieved facet, determine whether the input contains evidence that supports the facet, contradicts the facet, or provides insufficient evidence to establish either relation.

For each evaluated facet, return:

1. facet_id: the identifier of the corresponding retrieved ontology facet.
2. polarity: exactly one of support, contradict, or neutral.
3. intensity: an integer in $\left\{ 0 , 1 , 2 \right\}$, where 0 denotes absent or insufficient evidence, 1 denotes present/moderate evidence, and 2 denotes strong or explicit evidence.
4. quote: a verbatim supporting or contradicting span from the input text, limited to a maximum of 25 words. Do not fabricate or paraphrase quotations.
5. rationale: a concise explanation linking the quoted textual evidence to the retrieved symptom-facet definition.

**S1.4 Evidence-Extraction Constraints**

Apply the following constraints:

- Use only information contained in {INPUT_SEGMENT} when deciding whether symptom evidence is present.
- Use the retrieved ontology information only to define and disambiguate the candidate symptom facets; do not use it as evidence that the subject exhibits a symptom.
- Do not assign any facet_id that is absent from {RETRIEVED_FACETS}.
- Assign support only when the input segment contains evidence consistent with the retrieved facet.
- Assign contradict when the segment explicitly denies or contains evidence inconsistent with the retrieved facet.
- Assign neutral with intensity 0 when the available text is insufficient to support or contradict the facet.
- Do not infer symptoms from demographic characteristics, general context, or information that is not explicitly represented in the segment.
- The quote must reproduce the source text verbatim and must not exceed 25 words.
- Do not generate a depression diagnosis, depression probability, PHQ score, treatment recommendation, or screening decision.
- Preserve supporting and contradictory evidence when both occur so that contradiction can be represented by the downstream aggregation module.
- Return only valid JSON conforming to the schema specified below.

The contradiction rule is important because the manuscript explicitly models contradiction as a downstream evidence feature.

**S1.5 Required JSON Output Schema**

Use:

{

"evidence": [

{

"facet_id": "<retrieved facet identifier>",

"polarity": "<support|contradict|neutral>",

"intensity": 0,

"quote": "<verbatim quotation of no more than 25 words>",

"rationale": "<brief evidence-grounded explanation>"

}

]

}

**Field constraints**

| Field | Allowed content |
| --- | --- |
| facet_id | Identifier contained in the retrieved facet set |
| polarity | support, contradict, or neutral |
| intensity | Integer: 0, 1, or 2 |
| quote | Verbatim input-text span, maximum 25 words |
| rationale | Concise explanation grounded in the input and retrieved facet definition |

These five elements correspond directly to those already defined by R-GEE in Section 4.2.

**S1.6 Handling Insufficient and Contradictory Evidence**

When the input segment contains insufficient evidence for a retrieved symptom facet, the model assigns polarity = "neutral" and intensity = 0 rather than inferring symptom presence from contextual or external information. When the text explicitly contradicts the symptom definition, polarity = "contradict" is assigned and the corresponding textual span is retained. Supporting and contradictory observations are preserved as structured evidence items so that the downstream aggregation stage can compute contradiction scores and temporal consistency across segments. This separation between evidence extraction and downstream prediction prevents the LLM from directly determining the final depression label and preserves the modular design of R-GCRED.

That last point is methodologically important: the manuscript explicitly states that the LLM is not being used as an end-to-end labeler.

**S1.7 Output Validation and Regeneration**

Each LLM response is parsed and checked against the predefined JSON schema before being accepted by the evidence-aggregation module. A response is considered invalid if required fields are missing, facet_id does not correspond to the retrieved candidate set, polarity falls outside the permitted categories, intensity falls outside the interval $\left\{ 0 , 1 , 2 \right\}$, the response cannot be parsed as valid JSON, or the output otherwise violates the predefined structural constraints. Invalid outputs are discarded and the extraction request is regenerated using the same input segment, retrieved ontology context, and prompt template. Only schema-conforming outputs are admitted to the subsequent evidence-aggregation stage.
